# Exploring the Underlying Mechanism of Weiling Decoction Alleviates Cold-Dampness Diarrhea Based on Network Pharmacology, Transcriptomics, Molecular Docking and Experimental Validation

**DOI:** 10.3390/ph18010109

**Published:** 2025-01-16

**Authors:** Yannan Zhang, Shuai Zhang, Yimeng Fan, Sijuan Huang, Shimin Wang, Zhihui Hao, Jianzhong Shen

**Affiliations:** 1 National Key Laboratory of Veterinary Public Health Security, College of Veterinary Medicine, China Agricultural University, Beijing 100193, China; b20193050423@cau.edu.cn (Y.Z.); b20243050538@cau.edu.cn (S.Z.); b20223050425@cau.edu.cn (Y.F.); b20223050398@cau.edu.cn (S.H.); 2 Key Biology Laboratory of Chinese Veterinary Medicine, Ministry of Agriculture and Rural Affairs, Beijing 100193, China; 3 College of Veterinary Medicine, Xinjiang Agricultural University, Urumqi 830052, China; smwang@xjau.edu.cn

**Keywords:** weiling decoction (WLD), cold-dampness diarrhea (CDD), Traditional Chinese Medicine, CD4+ T-cell balance, gut barrier

## Abstract

**Background**: Cold-dampness diarrhea (CDD) is a common gastrointestinal disorder in children, characterized by diarrhea and intestinal barrier dysfunction. Weiling decoction (WLD) is frequently used in clinical practice to treat CDD, a condition triggered by multiple factors. However, the molecular mechanisms underlying its therapeutic effects remain poorly understood. **Objectives**: This study aimed to evaluate the efficacy of WLD in treating CDD and to elucidate its potential mechanisms. **Methods**: UPLC-HRMS/MS was employed to identify the chemical constituents of WLD and the absorption components in the plasma of WLD-treated rats. Additionally, a rat model of CDD was established to assess the therapeutic effects of WLD through a comprehensive approach. To elucidate the molecular mechanisms underlying these effects, network pharmacology and transcriptomic analyses were performed to identify potential signaling pathways associated with CDD alleviation. Molecular docking and flow cytometry assays were subsequently utilized to validate the identified signaling pathways. **Results**: A total of 223 chemical components were detected in WLD, and 49 absorption components were identified in the plasma of WLD-treated rats by UPLC-HRMS/MS. WLD treatment significantly alleviated the symptoms of CDD, reduced intestinal damage, and diminished the inflammatory response. Additionally, WLD influenced key genes in immune-related pathways. Molecular docking revealed strong binding affinities between the main components of WLD and key targets within these pathways. Flow cytometry, along with the analysis of inflammatory cytokines and transcription factors, demonstrated that WLD modulated the balance between Th1/Th2 and Th17/Treg cell populations. **Conclusions**: This study provides the first evidence that WLD alleviates CDD by regulating the balance between Th1/Th2 and Th17/Treg cell populations. These findings offer a theoretical basis for future investigations into the therapeutic potential of WLD in the treatment of CDD.

## 1. Introduction

Cold-dampness diarrhea (CDD) is a gastrointestinal disorder caused by a greasy, cold diet or exposure to cold and damp environments, characterized by recurrent diarrhea, abdominal pain, and impaired gastrointestinal function [1]. Common symptoms of CDD typically include loose stools, emaciation, weakness, and fatigue, and it is one of the most prevalent types of pediatric diarrhea [2,3]. The occurrence of CDD arises from the complex interaction of host-specific factors and environmental influences. Its pathogenesis is multifaceted, involving intestinal flora disruptions, activation of inflammatory responses, reactive oxygen species accumulation, immune dysregulation, and bacterial or viral infections [1,4,5]. These contributing factors collectively contribute to diarrhea, intestinal barrier dysfunction, and chronic inflammatory response [6]. Current clinical therapies, such as diosmectite and antibiotics, are effective in alleviating symptoms but are associated with potential adverse effects, particularly with long-term use. This underscores the urgent need to develop safer, more effective therapeutic strategies. Such efforts will not only deepen our understanding of CDD but also provide a robust foundation for designing novel treatments to combat this condition more effectively.

Traditional Chinese Medicine (TCM) has been widely used in Asian countries for thousands of years and offers unique advantages in treating intestinal diseases [7,8,9]. Among its diverse formulations, weiling decoction (WLD), a remedy derived from Danxi’s Experiential Therapy, is widely regarded as a classic prescription for treating CDD. Several scholars have demonstrated that WLD has significant efficacy in managing acute non-bacterial diseases, functional diarrhea, and acute childhood diarrhea, particularly during the fall season [10,11,12,13]. Moreover, recent research indicated that WLD exhibits notable antidiarrheal and immunomodulatory effects [14]. Additionally, WLD has been reported to ameliorate immune-associated mossy dermatitis in non-small-cell lung cancer [15]. Further studies in rat models of CDD revealed that WLD improves symptoms by enhancing urinary d-xylose excretion rates and increasing serum levels of gastrin (GAS) and motilin (MTL) [16]. Compared to diosmectite and antibiotics, WLD treatment offers distinct advantages, being both safe and free of significant side effects. Therefore, WLD has become one of the most promising therapeutic options for CDD. Despite existing evidence supporting WLD’s therapeutic potential for CDD, its efficacy remains insufficiently validated. Furthermore, the precise molecular mechanisms underlying WLD’s therapeutic effects in treating CDD are yet to be elucidated.

Emerging studies suggest that the pathogenesis of diarrhea and colitis may be related to an imbalance in the CD4+ T-cell subpopulation [17]. Maintaining equilibrium between Th1/Th2 and Th17/Treg cells is crucial, as it alleviates excessive immune response in colitis and restores homeostasis between the intestinal mucosal and microbiota [18]. When Th1 is overly activated, it may lead to inflammatory bowel disease and other conditions, whereas Th2 dominance is associated with allergic reactions [19]. Abnormalities in T-cells, such as a reduced or absent Treg cell population, can lead to immune imbalance, resulting in immune disorders and sustained inflammatory responses in the gut. Cytokines and transcription factors associated with Th1/Th2 and Th17/Treg cells are critical for suppressing proinflammatory cytokines, such as those derived from Th1 and Th17 cells, thus mitigating intestinal inflammation [20]. Therefore, targeting the balance of Th1/Th2 and Th17/Treg cells represents a promising therapeutic approach for managing CDD. However, no studies to date have specifically examined whether WLD exerts therapeutic effects on CDD through modulation of Th1/Th2 and Th17/Treg cell balance.

However, while WLD has demonstrated significant clinical efficacy against CDD, most existing studies have focused primarily on assessing its overall therapeutic effects, with limited attention given to understanding its underlying mechanisms of action. To address this gap, we establish a robust CDD rat model to systematically investigate the therapeutic effects of WLD. In contrast to previous work, our study employs a multifaceted approach that integrates network pharmacology, transcriptomic analysis, molecular docking, and flow cytometry, providing a comprehensive evaluation of WLD’s mechanisms. This investigation uniquely reveals, for the first time, the pivotal role of WLD in modulating the Th1/Th2/Th17/Treg immune balance. By elucidating its molecular targets and associated signaling pathways, our findings not only advance the understanding of WLD’s pharmacological effects but also provide novel insights into its molecular mechanisms in mitigating CDD.

## 2. Results

### 2.1. Identification of the Main Chemical Components in WLD by UPLC-MS/MS

**Figure 1:**
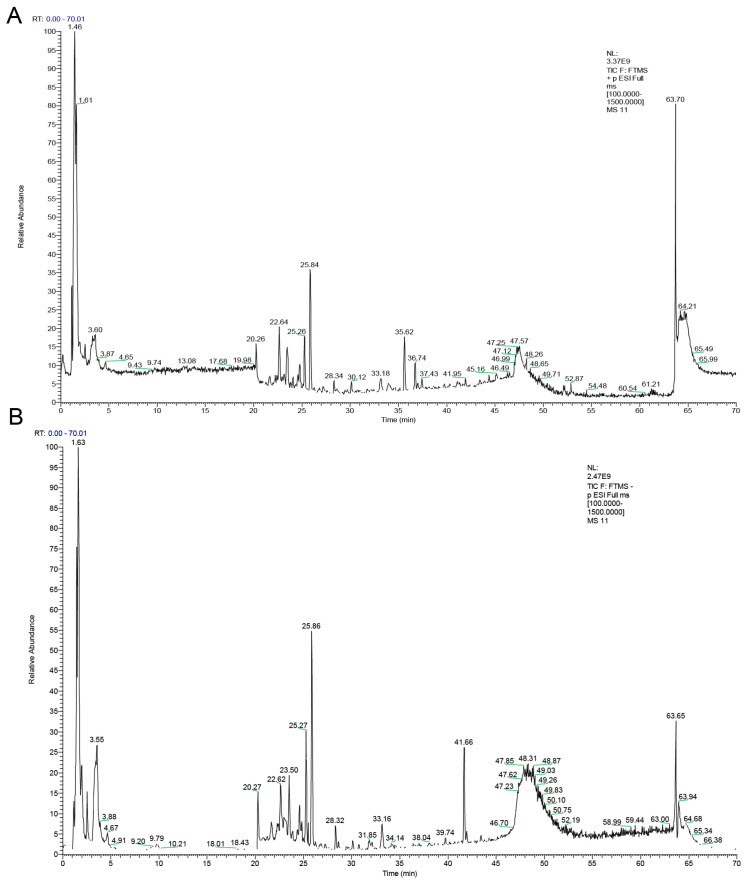
Total ion chromatograms of WLD by UHPLC-MS/MS. (**A**) Positive ion mode in the total ion flow diagram. (**B**) Negative ion mode in the total ion flow diagram.

To identify the main chemical components in WLD, we employed UPLC-MS/MS analysis, and the total ion flow chromatograms are displayed in both positive and negative ion modes (Figure 1A,B). A total of 223 main chemical compounds were identified, which included 66 flavonoids, 50 organic acids, 25 miscellaneous compounds, 21 terpenoids, 14 glycosides, 13 phenols, 9 amino acids, 8 phenylpropanoids, 7 alkaloids, 5 lactones, 2 lignans, 2 quinones, and 1 nucleotide (Table S2). These findings highlighted the extensive chemical diversity of WLD, providing a solid foundation for subsequent network pharmacology analyses. In addition, we quantified seven key markers, including (1) Hesperidin, (2) Atractylenolide-III, (3) Honokiol, (4) Magnolol, (5) Atractylenolide-II, (6) Atractylenolide-I, and (7) Alisol B 23-acetate, using validated analytical methods to determine their absolute concentrations (Figure S1A,B). Their structures were confirmed through secondary mass spectrometry (Figure S1C–I). The calibration curves for these markers exhibited good linearity, with regression coefficients (R) greater than 0.99 (Figure S2). A summary of the relevant parameters is provided in Table S1.

### 2.2. Validation of the CDD Model Rats and Therapeutic Effects of WLD

**Figure 2:**
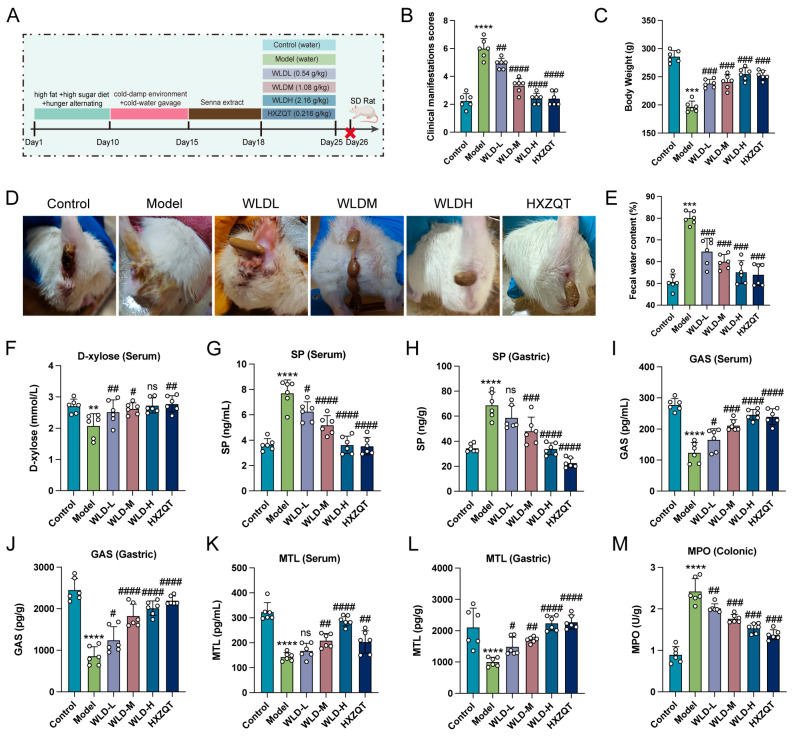
The protective effect of WLD on CDD rats. (**A**) A schematic diagram illustrates the experimental course in which WLD was used to treat CDD model rats. (**B**) Clinical symptom scores were evaluated in different groups (n = 6). (**C**) Body weight changes were recorded in different groups (n = 6). (**D**) The incidence of diarrhea was assessed in different groups (n = 6). (**E**) Fecal water content was measured in different groups (n = 6). (**F**) D-xylose levels in rat serum were analyzed across different groups. (**G**–**L**) Levels of various gastrointestinal hormones (SP, GAS, and MTL) in serum and gastric tissue were measured across different groups (n = 6). (**M**) MPO levels in colonic tissue were compared among different groups (n = 6). ** *p* < 0.001, *** *p* < 0.001, and **** *p* < 0.001 compared to the control group, and # *p* < 0.05, ## *p* < 0.01, ### *p* < 0.001, and #### *p* < 0.001 compared to the model group (ns: *p* > 0.05).

To validate the CDD model, clinical symptoms and associated indicators were systematically assessed (Figure S3A). The results demonstrated that CDD rats exhibited symptoms such as loose stools, reduced activity, disheveled fur, and hair loss, resulting in a high symptom score (Figure S3B). Additionally, there was a loss of body weight (Figure S3C) and an increase in fecal water content (Figure S3D). Following WLD administration, the clinical symptom score decreased, body weight increased significantly, and fecal water content decreased significantly (*p* < 0.001; Figure 2A–E). To determine the effect of WLD on intestinal absorption function, we administrated D-xylose and detected the levels of D-xylose in the serum. The results showed that WLD treatment increased D-xylose levels, suggesting that WLD promoted the intestinal absorption function (Figure 2F). Furthermore, we detected the levels of gastrointestinal hormones, such as substance P (SP), GAS, and MTL in the serum and gastric tissues, which are associated with intestinal regulation function. The results demonstrated that WLD treatment significantly decreased SP levels while increasing GAS and MTL levels (*p* < 0.05; Figure 2G–L), indicating that WLD regulates the imbalance of gastrointestinal hormones. In addition, we evaluated the anti-inflammatory capacity in CDD rats treated with WLD. WLD treatment significantly reduced myeloperoxidase (MPO) levels in the colon, indicating that WLD attenuated the intestinal inflammatory response in CDD rats (*p* < 0.01; Figure 2M). In conclusion, WLD administration mitigated CDD symptoms by restoring the gastrointestinal hormone balance, reducing inflammation responses.

### 2.3. WLD Protects Intestinal Barrier Integrity by Enhancing Tight Junctions and Restoring Epithelial Function in CDD Rats

**Figure 3:**
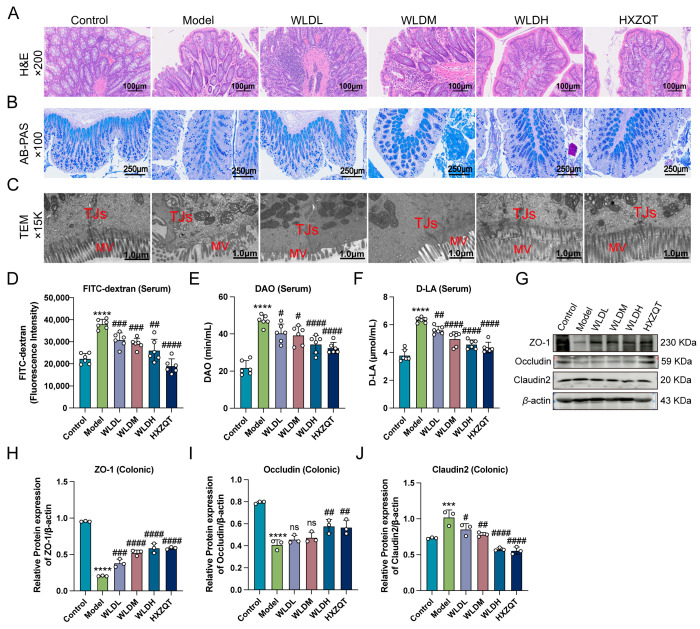
The alleviating effect of WLD on intestinal barrier damage. (**A**) Pathological changes in the colon of CDD rats were examined using the H&E staining method across different groups. Scale bar: 100 μm; original magnification: ×200. (**B**) Goblet cell populations in different groups were assessed using the AB-PAS staining method. Scale bar: 250 μm; original magnification: ×100. (**C**) The structures of microvilli and tight junctions were examined in different groups using transmission electron microscopy (TEM). Scale bar: 1.0 μm; original magnification: ×15 K. (**D**) FITC-dextran levels in rat serum were quantified across different groups (n = 6). (**E**,**F**) Various permeability indicators (D-AO and D-LA) were assessed in rat serum from different groups (n = 6). (**G**) Expression levels of different tight junction proteins were detected by Western blotting (WB). (**H**–**J**) The quantified analysis of different tight junction proteins was measured in different groups (n = 3). *** *p* < 0.001 and **** *p* < 0.001 compared to the control group, and # *p* < 0.05, ## *p* < 0.01, ### *p* < 0.001, and #### *p* < 0.001 compared to the model group (ns: *p* > 0.05).

To determine the protective effect of WLD against intestinal barrier damage in CDD rats, we observed changes in the intestinal structure using hematoxylin and eosin (H&E), alcian blue–periodic acid-Schiff (AB-PAS) staining, and transmission electron microscopy (TEM). H&E staining revealed that the model group exhibited crypt damage, epithelial layer defects, and significant mucosal injury. In contrast, WLD treatment significantly ameliorated intestinal damage and restored epithelial cell integrity in CDD rats (Figure 3A). Similarly, AB-PAS staining showed that the number of goblet cells in the colon was significantly reduced in the CDD group, while WLD treatment increased the number (Figure 3B). Additionally, TEM analysis of colonic tissues showed that the model group had irregular and fragmented microvilli, a sharp reduction in microvilli density, and widened tight junctions between cells. Conversely, the microvilli in the WLD-treated group were neatly and densely arranged, resembling the normal structure (Figure 3C). Moreover, intestinal permeability assays further demonstrated that WLD treatment significantly reduced the serum levels of FITC-dextran, DAO, and D-LA in CDD rats (*p* < 0.01), suggesting that WLD effectively reduced intestinal permeability (Figure 3D–F). Western blot analysis revealed that the expression levels of tight junction proteins, including ZO-1 and Occludin, were significantly reduced in the model group, but WLD treatment reversed these effects. Additionally, Claudin-2 protein expression exhibited an opposite trend (Figure 3G–J). In summary, these results indicated that WLD exerted a protective effect on intestinal barrier function in CDD rats by restoring epithelial integrity and mucosal permeability and enhancing tight junction protein expression.

### 2.4. WLD Alleviated CDD Symptoms Through Network Pharmacology and CD4+ T-Cell Pathway Analysis

**Figure 4:**
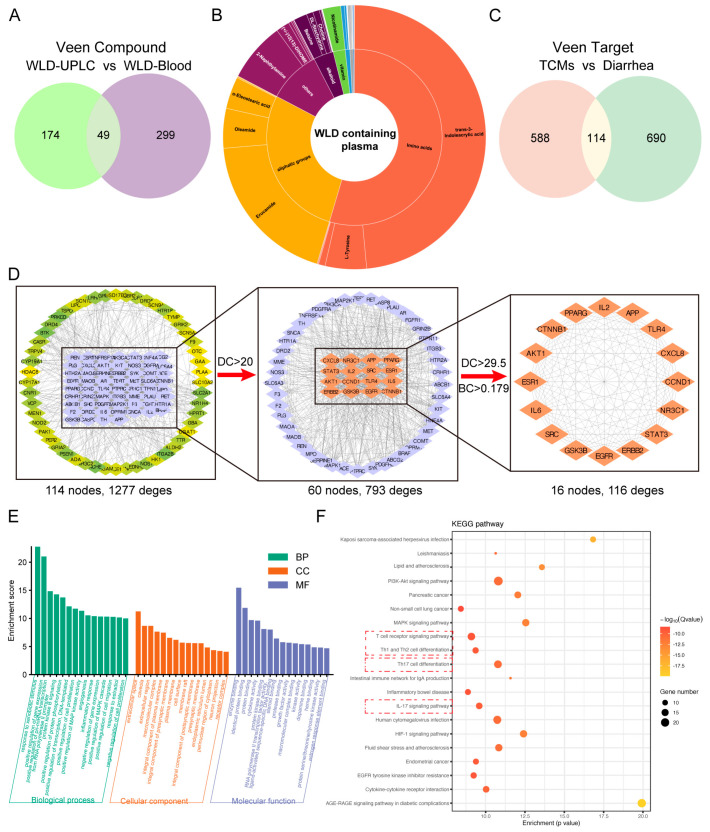
The alleviation of CDD by WLD administration is related to the balance of Th1/Th2 and Th17/Treg cells. (**A**) Potential key components associated with CDD were identified by intersecting the WLD components and the blood-entry components. (**B**) These potential key components were classified. (**C**) Key targets related to CDD were determined by intersecting the key component targets of WLD with the potential disease targets. (**D**) Key targets were selected with DC and BC from the PPI network. (**E**) GO enrichment analysis was presented as WLD against CDD. (**F**) The top 20 signaling pathways were enriched by KEGG pathways.

To investigate the components in WLD that may contribute to alleviating CDD, we administered WLD to the model rats and employed UPLC-MS/MS technology to analyze the plasma chemistry. This approach identified 348 components (Table S3), 49 of which were selected for further comparison with WLD’s mass spectrometry results and subsequent analysis (Figure 4A). These selected components were grouped into the following categories: alkaloids (9, 18.37%), terpenoids (8, 16.33%), organic acids (6, 12.24%), amino acids (5, 10.20%), fatty acids (5, 10.20%), phenols (4, 8.16%), flavonoids (1, 2.04%), vitamins (1, 2.04%), phenylpropanoids (1, 2.04%), polysaccharides (1, 2.04%), and others (8, 16.33%) (Figure 4B). A PPI network was constructed using 114 potential targets, which were identified as the intersection of 702 component-related targets and 804 disease-related targets (Figure 4C). This network, comprising 114 nodes and 1277 edges, was generated using the STRING database, and subsequently analyzed to identify core targets. Median values for degree centrality (DC), betweenness centrality (BC), and closeness centrality (CC) were 29.5, 0.179, and 0.5586, respectively. Thresholds were set at values greater than or equal to the median for these parameters. Using these criteria to prioritize key targets and interactions, the analysis was narrowed down to 16 nodes and 116 edges (Figure 4D). Among the most pivotal targets identified were genes such as IL-2, TLR4, EGFR, IL-6, and AKT1. These genes are known to play critical roles in the development and progression of diarrhea-related diseases. To explore the functional roles of these targets, GO and KEGG enrichment analyses were performed using the DAVID database. GO enrichment analysis revealed that WLD primarily influenced biological processes (BP), cellular components (CC), and molecular function (MF), such as positive regulation of gene expression, protease B signaling, protein phosphorylation, inflammatory response, cell proliferation feedback, neuroprotection, receptor complex formation, enzyme activity, cytokine activity, and protein binding (Figure 4E). Furthermore, Kyoto Encyclopedia of Genes and Genomes (KEGG) pathway enrichment analysis showed that WLD modulated several key signaling pathways, including T-cell receptor signaling, Th1/Th2 cell differentiation, Th17 cell differentiation, IL-17, PI3K-AKT, MAPK, and HIF-1 signaling (Figure 4F). Together, these findings suggest that WLD exerted a protective effect by regulating key pathways, particularly those involved in T-cell receptor signaling, Th1/Th2 cell differentiation, and Th17 cell differentiation.

### 2.5. Th1/Th2 and Th17/Treg Pathways Play Key Roles in WLD-Mediated Alleviation of CDD

**Figure 5:**
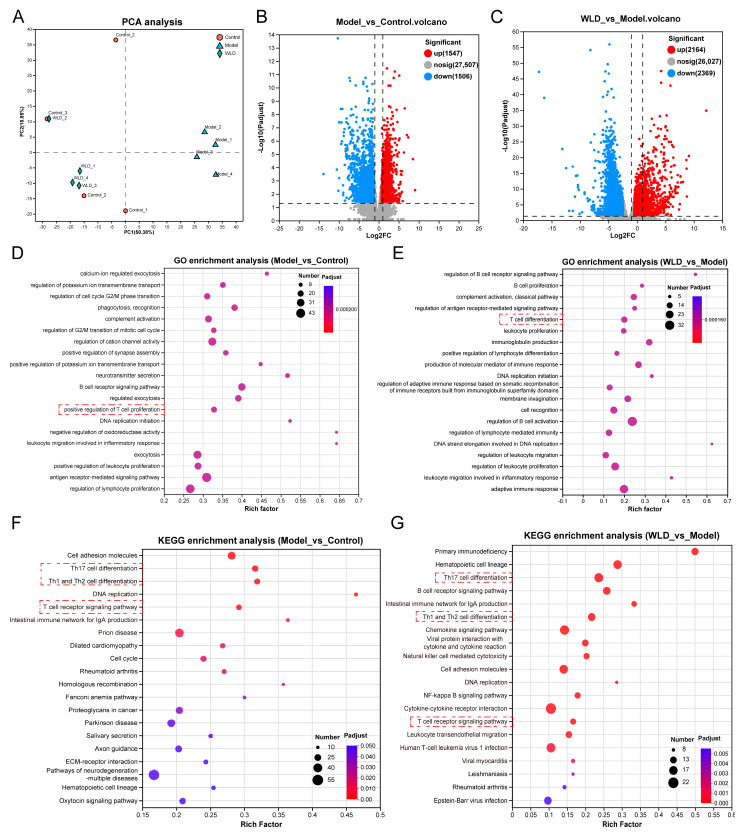
Transcriptomic analysis of the potential mechanism of WLD in alleviating CDD. (**A**) PCA to reflect the alteration with WLD in CDD. (**B**,**C**) Differentially expressed genes were compared between the model group vs. control group and WLD vs. model group (n = 4). (**D**,**E**) GO enrichment analysis between model group vs. control group and WLD vs. model group (n = 4). (**F**,**G**) KEGG pathways were enriched based on comparisons of the control group vs. model group and WLD vs. model group (n = 4).

To further investigate the mechanism of CDD alleviation by WLD, we performed transcriptomic sequencing of colonic tissues. Principal component analysis (PCA) revealed a significant separation between the model group and the other two groups, while some overlap was noted between the control group and the WLD administration group. This partial overlap indicates that WLD had a notable alleviating effect on CDD (Figure 5A). Comparative analysis indicated that in the model group, 1547 genes were upregulated and 1506 genes were downregulated compared to the control group (Figure 5B). In contrast, the WLD group showed 2164 upregulated genes and 2369 downregulated genes relative to the model group (Figure 5C). GO enrichment analysis revealed that WLD influenced several biological processes, including T-cell differentiation, DNA replication proliferation, antigen-receptor-mediated signaling pathway, adaptive immune response, and B-cell activation regulation (Figure 5D,E). Furthermore, KEGG pathway enrichment analysis revealed significant alterations in pathways related to Th17 cell differentiation, Th1 and Th2 cell differentiation, and T-cell receptor signaling when comparing the WLD group to the model and control groups (Figure 5F,G). Additionally, differentially expressed genes showed that WLD affected Th1/Th2 cell pathway-related genes (Nfatc2, Lck, Lat, Cd247, Cd3d, IL2ra, and IL2rb; Figure S4A,B), the Th17 cell pathway-related genes (Nfatc2, Irf4, IL27ra, Lck, Lat, Cd247, Cd3d, IL2ra, and IL2rb; Figure S4C,D), and the T-cell receptor pathway-related genes (Lat, Lck, Cd247, Cd3d, and Nfatc2; Figure S4E,F). For example, Lat and Lck play crucial roles in T-cell maturation and signaling. Cd247 and Cd3d are expressed on the surface of T lymphocytes, where they play a major role in adaptive immunity. Additionally, the Nfatc2 gene is involved in cytokine expression by T-cells. In conclusion, these genes are crucial for T-cell differentiation, maturation, and signal transduction. The pathway analysis revealed significant enrichment in signaling pathways related to T-cell differentiation (Th1/Th2 and Th17/Treg cell) following WLD administration. These findings suggest that WLD, to a certain extent, modulated intestinal immunity by influencing immune function, thereby alleviating CDD.

### 2.6. WLD Components Target Th1/Th2 and Th17/Treg Pathways in CDD Rats

**Figure 6:**
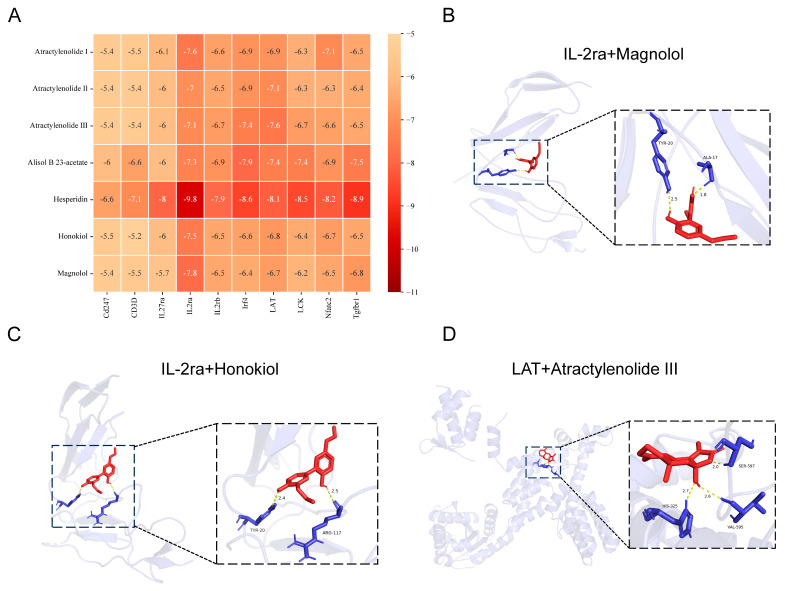
Molecular docking of representational components in WLD with key targets in the Th1/Th2 and Th17/Treg pathways. (**A**) Post-docking binding energy thermograms for seven components of WLD with ten key proteins in the Th1/Th2 and Th17/Treg signaling pathways. (**B**) Molecular docking of IL-2ra with Magnolol. (**C**) Molecular docking of IL-2ra with Honokiol. (**D**) Molecular docking of LAT with Atractylenolide-III.

To confirm the role of WLD in alleviating CDD through the Th1/Th2 and Th17/Treg signaling pathways, we performed molecular docking analysis on seven representative components of WLD to elucidate their interactions with key proteins. Figure 6A presents the binding energies (kcal/mol) of the main targets and seven representative components. The docking results revealed that all free-binding energies ranged from −5.2 to −9.8 kcal/mol, with scores lower than −5 kcal/mol, indicating high binding affinities for all interactions. Quantitative analysis identified three of the seven components—Magnolol, Honokiol, and Atractylenolide III—as present in high concentrations, exceeding 100 μg/g (Table S4). The free-binding energies of Magnolol and Honokiol with IL2ra were −7.8 kcal/mol and −7.5 kcal/mol (Figure 6B,C), respectively, while that of Atractylenolide III with Lat was −7.6 kcal/mol (Figure 6D). Docking scores for all three components were below −7 kcal/mol, further supporting their strong binding affinities with IL2ra and Lat. These findings suggest that WLD components interacted with key molecules involved in the Th1/Th2 and Th17/Treg signaling pathways, underscoring WLD’s therapeutic potential for the treatment of CDD.

### 2.7. WLD Administration Alleviated CDD Symptoms by Restoring Th1/Th2 and Th17/Treg Cell Balance

**Figure 7:**
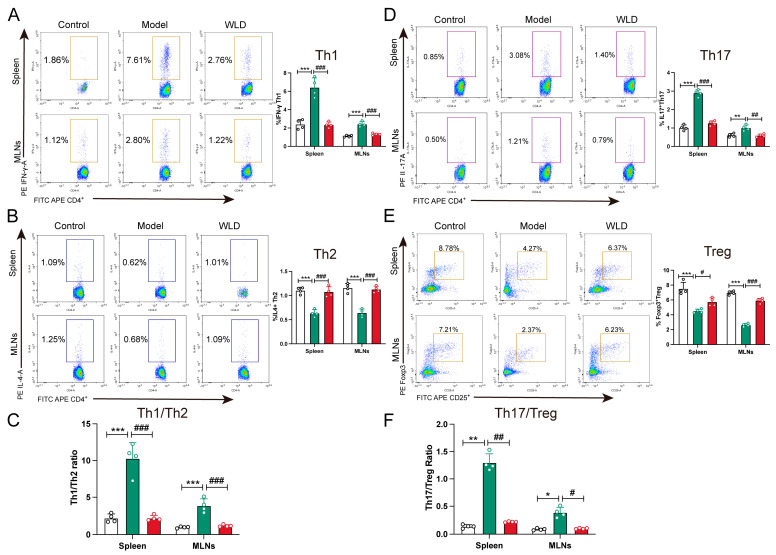
WLD treatment regulates the ratio of Th1/Th2 and Th17/Treg in the spleen and mesenteric lymph nodes (MLNs). (**A**,**B**) The cell populations of Th1 and Th2 in different groups were detected by flow cytometry (n = 4). (**C**) The Th1/Th2 cell ratio was analyzed using GraphPad software (n = 4). (**D**,**E**) The cell populations of Th17 and Treg in different groups were also detected by flow cytometry (n = 4). (**F**) The Th17/Treg cell ratio was analyzed using GraphPad software (n = 4). * *p* < 0.05, ** *p* < 0.01, and *** *p* < 0.001 compared to the control group, and # *p* < 0.05, ## *p* < 0.01, and ### *p* < 0.001 compared to the model group. White column was control group, green column was model group, and red column was WLD group.

**Figure 8:**
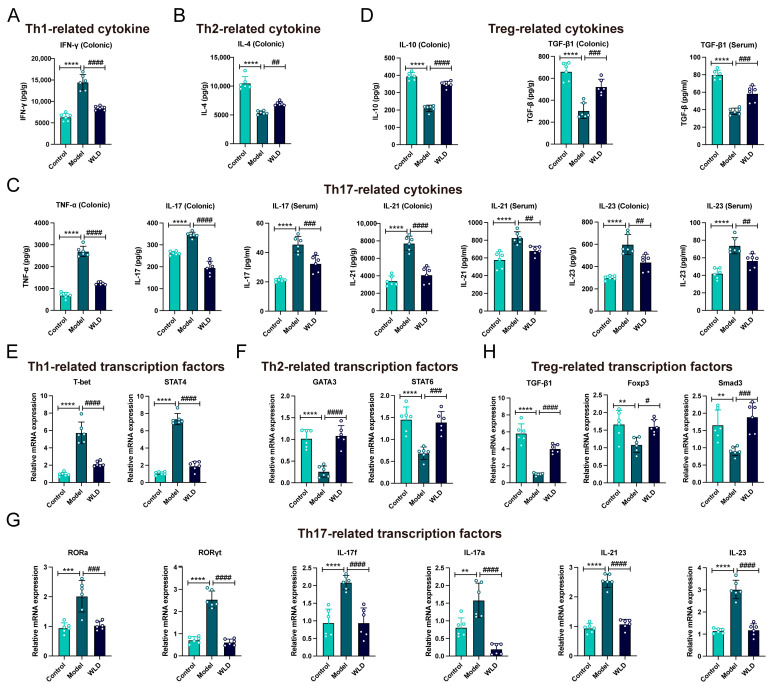
WLD regulated the levels of inflammatory factors and transcription factors associated with Th1/Th2 and Th17/Treg cell differentiation. (**A**,**B**) The levels of Th1- (IFN-γ) and Th2-related inflammatory factors (IL-4) in serum were measured across different groups (n = 6). (**C**) Th17-related inflammatory factors (TNF-α, IL-17, IL-21, and IL-23) were quantified in both serum and colonic tissue in different groups (n = 6). (**D**) Treg-related inflammatory factors (IL-10 and TGF-β) were assessed in serum and colonic tissue in different groups (n = 6). (**E**,**F**) Levels of Th1/Th2-related transcription factors (T-bet, STAT4, GATA3, and STAT6) were measured in colonic tissue across different groups (n = 6). (**G**) Th17-related transcription factors (RORa, RORγt, IL-17f, IL-17a, IL-21, and IL-23) were quantified in colonic tissue in different groups (n = 6). (**H**) Treg-related transcription factors (TGF-β1, Foxp3, and Smad3) were quantified in colonic tissue in different groups (n = 6). ** *p* < 0.01, *** *p* < 0.001, and **** *p* < 0.0001 compared to the control group, and # *p* < 0.05, ## *p* < 0.01, ### *p* < 0.001, and #### *p* < 0.0001 compared to the model group.

As previously reported, the dynamic balance between CD4+ T-cell subsets influence the onset and progression of diarrhea and inflammation during its course [21]. To assess the effect of WLD on the Th1/Th2 balance, we conducted flow cytometry to analyze the populations of these cell types in the spleen and mesenteric lymph nodes (MLNs). Our findings indicated that WLD treatment significantly downregulated Th1 cell populations while upregulating Th2 cell numbers, thereby restoring the Th1/Th2 balance compared to the model group (Figure 7A–C). Similarly, the Th17/Treg balance was assessed, and it was found that WLD significantly reduced the number of Th17 while increasing the number of Treg cells, thus restoring the balance between the Th17/Treg cells (*p* < 0.05; Figure 7D–F). To further validate these findings, we measured the serum and colonic levels of various cytokines, including Th1-associated cytokine IFN-γ, Th2-related cytokine IL-4, as well as Th17-associated cytokines TNF-α, IL-17, IL-21, and IL-23, and Treg-associated cytokines IL-10 and TGF-β1. The results indicated that WLD administration significantly reduced the levels of the Th1-associated cytokine IFN-γ and Th17-associated cytokines (TNF-α, IL-17, IL-21, and IL-23), while it significantly elevated the levels of the Th2-related cytokine IL-4 and Treg-associated cytokines (IL-10 and TGF-β1) in the CDD group (*p* < 0.01). These findings were consistent with our previous conclusions derived from flow cytometry and transcriptomics (Figure 8A–D). Furthermore, transcription factors, as key drivers of T-cell differentiation, are closely associated with various immune-related diseases [22]. To investigate this further, we analyzed the Th1/Th2-related transcription factors T-bet, STAT4, GATA3, and STAT6, as well as the Th17/Treg-related transcription factors RORα, RORγt, and IL-17f. The results demonstrated that WLD treatment significantly decreased the levels of Th1- and Th17-related transcription factors in the model group, while significantly increasing the levels of Th2- and Treg-related transcription factors (*p* < 0.05). These findings further corroborate our previous results (Figure 8E–H). Taken together, these results suggest that WLD enhanced intestinal mucosal immunity in rats with CDD by restoring the balance of Th1/Th2 and Th17/Treg cell ratios.

## 3. Discussion

CDD is one of the main types of pediatric diarrhea, which mainly impairs absorption in children, causing growth and developmental delays, imposing a significant burden on families and patients [1,3,23]. However, the pathogenesis mechanism for CDD is unclear, which seriously impedes CDD research. Previous studies suggested that WLD strengthens the spleen and harmonizes the middle, induces diuresis and removes dampness, and is often used to treat CDD, pediatric non-bacterial infectious diarrhea, pediatric autumn diarrhea, skin urticaria, and other internal and surgical diseases [5,24]. In this study, we investigated the potential mechanisms of WLD in the treatment of CDD through network pharmacology analysis, transcriptomics, and in vivo experiments. Our findings demonstrated that WLD reduces the systemic inflammatory response, regulates gastrointestinal hormone imbalances, upregulates the expression of tight junction proteins, and restores the balance of Th1/Th2 and Th17/Treg cells. Additionally, WLD was shown to modulate Th1/Th2 and Th17/Treg cell-related cytokines and transcription factors, thereby alleviating the symptoms of cold-dampness diarrhea.

It has become a prevailing research trend to analyze the mechanism of action of Traditional Chinese Medicine (TCM) components through the integration of multiple complementary methods. As an emerging discipline, network pharmacology leverages its network-based approach to investigate the therapeutic effects of TCM compounds, capitalizing on their characteristics of multi-ingredient, multi-target, and multi-pathway synergy [25,26,27]. The disease prevention strategies of TCM are rooted in a systemic and holistic perspective on medication, where multiple components act synergistically to enhance the efficacy of treatment, primarily through blood components [28,29]. Serum pharmacochemistry research of TCM plays a crucial role in elucidating the absorption and metabolic process of drugs in vivo and has become an essential method for studying the material basis of TCM efficacy [30]. With the integration of network pharmacology, serum pharmacochemistry provides an effective means to uncover the potential mechanisms of active ingredients present in the blood, serving as a powerful tool for understanding the mechanisms of TCM in disease therapy [31]. To explore the potential mechanisms of WLD in the treatment of CDD, this study employed a combination of network pharmacology, serum absorption component analysis, and transcriptomics to identify enriched signaling pathways. The results revealed that, following WLD administration, the enriched signaling pathways were predominantly associated with T-cell differentiation. Consequently, pathways related to T-cell differentiation were further selected for in-depth investigation. This conclusion was further supported by the analysis of cytokines and transcription factors, confirming that WLD modulated the immune response to alleviate diarrhea. These findings align with previous research demonstrating the immunomodulatory effects of herbal medicines in treating gastrointestinal disorders [19,32,33]. The results showed that the differentially expressed genes primarily included Nfatc2, Irf4, IL27ra, Lck, Lat, Cd247, Cd3d, IL2ra, IL2rb, and Tgfbr1. Among these, Nfatc2 plays a critical role in regulating the expression of Th1 and Th2 cells. In Th1 cells, the IL-21 promoter inhibits IL-21 transcription by preventing the binding of NFATc2 to the promoter. Conversely, in Th2 cells, NFATc2 directly binds to the IL-21 promoter and activates its transcription. Furthermore, in Th1 cells, T-bet suppresses IL-21 transcription by inhibiting the binding of NFATc2 to the promoter [34]. Lat (linker for activation of T-cells) is a membrane adaptor protein that is essential for T-cell receptor (TCR) signaling. TCR signaling is initiated with the involvement of tyrosine kinases Lck and ZAP70, which phosphorylate the transmembrane adaptor LAT, thereby activating T-cells [35]. Additionally, the lymphocyte-specific protein tyrosine kinase (LCK) plays a pivotal role in TCR signal transduction, T-cell differentiation, and activation [36,37]. The subsequent molecular docking results revealed that the binding energies of the seven representative components in WLD to key targets were all less than –5 kcal/mol. This finding indicates that the components of WLD may alleviate the symptoms of CDD by modulating the immune response of T-cells. Moreover, the study demonstrated that molecular dynamics simulations can provide a more realistic representation of the binding capabilities [37]. Therefore, future studies should further validate these findings using molecular dynamics tests.

To further investigate the therapeutic effects of WLD on CDD, we performed colon transcriptome sequencing on WLD-treated rats. KEGG pathway enrichment analysis revealed significant activation of immune regulation signaling pathways, predominantly enriched in CD4+ T-cells. These findings align with results from network pharmacology and serum pharmacochemistry, further supporting the role of WLD in modulating immune responses. T lymphocytes are central to regulating intestinal immunity and influence the onset and progression of intestinal diseases [38,39,40]. T-cells differentiate into CD4+ or CD8+ cells, with the CD4+ subpopulation further differentiating into Th1, Th2, Th17, and Tregs under the function of different cytokines [41,42]. Th1 cells secrete proinflammatory cytokines [43], while Th2 cells are anti-inflammatory cells. The balance of Th1/Th2 cells is key to the body’s inflammation process [44]. Similarly, Th17 and Treg cells maintain a dynamic equilibrium, with their dysregulation—such as Treg reduction or Th17/Treg imbalance—linked to intestinal disease development [45,46,47,48,49,50,51,52]. Transcription factors associated with Th1, Th2, Th17, and Treg cells also play essential roles in immune regulation and humoral immunity [44,53,54,55,56]. Our results showed that WLD treatment significantly decreased the ratio of both Th1/Th2 and Th17/Treg cells in the spleen and mesenteric lymph nodes with CDD rats, thereby mitigating diarrhea.

The synthesis and secretion of interleukins are tightly regulated by immune or non-immune cell stimulation. INF-γ and TNF-α, secreted by Th1 cells, promote inflammatory responses, while IL-4 and IL-10, secreted by Th2 cells, exhibit anti-inflammatory effects. CDD increases Th1-related cytokines (INF-γ and TNF-α) and reduces Th2-related cytokines (IL-4 and IL-10) [57]. WLD administration significantly reduced INF-γ and TNF-α levels while increasing IL-4 and IL-10 levels, indicating that WLD effectively suppresses the inflammation caused by CDD. The Th17/Treg cell-mediated immune response is a key mechanism in the pathogenesis of diarrhea and inflammation [32,58]. Th17 cells drive inflammation by secreting cytokines, such as TNF-α, IL-17, IL-21, and IL-23 [59]. This study demonstrated elevated levels of Th17-related cytokines in the serum and colonic tissue of the model group, which were significantly reduced after WLD treatment. Conversely, Treg cells play a regulatory role by secreting anti-inflammatory cytokines IL-10 and TGF-β1. IL-10 inhibits Th1-related cytokine production by suppressing antigen-presenting cells, while TGF-β1 modulates antigen-specific T-cells to prevent colitis [50,60]. In the model group, Treg-related cytokines (IL-10 and TGF-β1) were significantly decreased, but their levels were restored after WLD administration. These findings indicate that WLD alleviated CDD by restoring the balance between Th1/Th2 and Th17/Treg cytokines, thereby mitigating immune dysregulation and inflammation.

Transcription factors play a pivotal role in regulating CD4+ T-cell differentiation. Th1 cells differentiate via the activation of transcription factors T-bet and STAT4 by IL-12 [19]. Reducing Th1 cell over-differentiation and the associated production of proinflammatory cytokines is critical for alleviating diarrhea. This study found that WLD significantly inhibited T-bet and STAT4 mRNA expression, thereby reducing inflammation responses and alleviating CDD. Th2 cells, activated by transcription factors GATA3 and STAT6, participate in humoral immunity and suppress Th1 differentiation by secreting IL-4. In the CDD model, the mRNA levels of GATA3 and STAT6 were significantly reduced, but WLD administration restored their expression. This restored the Th1/Th2 balance by reducing Th1 cell proportions and increasing Th2 cells. The regulatory effect of WLD on Th1/Th2-related cytokines and transcription factors aligns with Lv et al.’s findings, suggesting that WLD maintains immune homeostasis by modulating the differentiation of Th1 and Th2 cells, thereby alleviating diarrhea symptoms [18]. The differentiation of Th17 and Treg cells is regulated by distinct transcription factors. RORγt, the Th17-specific transcription factor, interacts with STAT3, which regulates Treg-to-Th17 conversion and suppresses Foxp3, the key transcription factor for Treg cells. This study showed that WLD significantly inhibited RORγt and STAT3 expression, counteracting Th17-mediated inflammation. Furthermore, TGF-β promotes Foxp3 expression via the Smad signaling pathway, enhancing Treg differentiation and suppressing RORγt activity [61]. This study found that WLD significantly upregulated the mRNA expression levels of Foxp3 and Smad3, thereby promoting Treg differentiation. These findings indicate that WLD alleviated CDD by restoring the Th1/Th2 and Th17/Treg balance through the regulation of key transcription factors and cytokines.

In summary, our study revealed that WLD may alleviate CDD by regulating the Th1/Th2 and Th17/Treg cell balance, thereby exerting therapeutic effects on CDD. This study further elucidated and expanded the understanding of WLD’s potential mechanisms in treating CDD. However, there are still some limitations in our research, particularly the need to validate the mechanisms through in vitro experiments. Additionally, subsequent studies can explore how specific blood-entry components in WLD regulate CD4+ T-cell homeostasis and contribute to the improvement of CDD symptoms.

## 4. Materials and Methods

### 4.1. WLD and Senna Leaf Preparation

The WLD consisted of herbs purchased from TongRenTang Co., Ltd. (Beijing, China), and detailed information is provided in Table S4. The herbs were sequentially decocted in 10-fold, 8-fold, and 6-fold water (*m*/*v*) for 1 h. The filtrates were collected and lyophilized into a powder under freeze-dried conditions.

Senna leaf was obtained from TongRenTang Co., Ltd. (Beijing, China), and soaked in hot water for 12 h, and the filtrate was concentrated to 1.0 g/mL under reduced pressure.

### 4.2. Animals

Male Sprague-Dawley rats (180 ± 20 g, 5~6-week-old, SPF grade) were obtained from SPF (Beijing) Biotechnology Co., Ltd. (Beijing, China; Certificate No. SCXK (Jing) 2019-0010). The animals were housed in a controlled environment (12 h light/dark cycle, 23 ± 2 °C temperature, and 50% ± 5% humidity) for one week. They were given ad libitum access to food and water during a one-week acclimatization period. This experiment was approved by the Laboratory Animal Ethics Committee of China Agricultural University (approval date: 15 November 2022; approval number: AW51112202-2-3).

### 4.3. CDD Rat Model Establishment and Evaluation

The CDD rat model was established with minor modifications based on existing literature [25,62,63], and the sample size was determined using Mead’s resource equation. The rats were randomly assigned into two groups: the control group and the model group. The rats in the model group were fasted for one day, then administered lard at a dose of 1 mL/100 g body weight along with adequate food on the following day, and their water was supplemented with 30% honey daily for 10 days. After this period, these rats were exposed to a cold and humid environment (4 ± 0.5 °C and 90 ± 2% humidity) for 8 h daily over 5 days, with a gavage of 4 °C ice water administered once each day. Additionally, senna extract was administered via gavage once daily for the next 3 days. The success of the CDD model was assessed based on several indicators, including behavioral condition, mental status, coat quality, fecal characteristics, appetite, and body weight. The model was considered successful when symptom scores exceeded four (two principal symptoms plus two accompanying symptoms) [64]. Further details on symptom scoring are provided in Table 1, and the details of the principal symptoms and accompanied symptoms are provided in the Supplementary Materials Table S5. The control group received a standard diet and an equivalent volume of sterile water, without any additional interventions. Finally, each group consisted of six rats.

### 4.4. WLD Administration

The rats were randomly divided into six groups (n = 6 per group): the control group (healthy rats received sterile water by gavage), the model group (CDD rats received sterile water by gavage), the WLD high-dose (CDD rats received 2.16 g/kg of WLD by gavage), medium-dose (CDD rats received 1.08 g/kg of WLD by gavage), and low-dose groups (CDD rats received 0.54 g/kg of WLD by gavage), and the HXZQT group (CDD rats received 0.216 g/kg of WLD by gavage). To establish the CDD model, all groups except the control group underwent the model establishment procedure. After confirming successful model establishment, the treatment groups (excluding the control and model groups) received WLD doses, as outlined above, for seven consecutive days, while the control and model groups were administered sterilized physiological saline. Throughout the experiment, body weights, fecal water content, and clinical symptoms were recorded. At the end of the experiment, all rats were anesthetized with isoflurane and euthanized, and serum and tissue samples were collected for further analysis. To ensure unbiased results, all procedures were conducted using the blind method.

According to the guidelines of Chinese Medicine Pharmacology Research Technology, the daily prescription dose of WLD for adults was 12 g/d. Using the standard rat-to- human dose conversion formula (human dose/70 kg × 6.3), the equivalent dose for rats was calculated as 1.08 g/kg/d, which corresponds to the medium-dosage group (WLDM group). To investigate dose-dependent effects, two additional dosage groups were established: a lower dose of 0.54 g/kg (half the medium dose, WLDL group) and a higher dose of 2.16 g/kg (double the medium dose, WLDH group).

### 4.5. Measurement of Fecal Water Content

After administration, fecal samples from each group were collected, weighed, and analyzed to determine their fecal water content, according to our previous report [9].

### 4.6. Intestinal Permeability Experiment

Rats were administered 60 mg/100 g of fluorescein isothiocyanate (FITC)-labeled dextran (FITC-D, molecular weight: 3000–5000; Sigma-Aldrich, St. Louis, MO, USA). Serum samples were obtained 4 h after administration, and fluorescence was detected using a CKX53 fluorescent microscope (Olympus, Tokyo, Japan) [65,66].

### 4.7. Measurement of Gastrointestinal Hormones and Biochemical and Inflammatory Indicators

SP, GAS, MTL, MPO, DAO, and D-LA levels were measured in serum and tissues using the corresponding kits, while IFN-γ, IL-4, IL-10, TGF-β1, TNF-α, IL-17, IL-21, and IL-23 levels were measured using the supernatant of colon tissue and serum, which were conducted with the manufacturer’s requirements (Shanghai Enzyme Linked Biotechnology Co., Ltd., Shanghai, China).

### 4.8. Histological Examination

Colon tissues were stained with hematoxylin and eosin (H&E; Solarbio, Beijing, China) and periodic acid-Schiff and alcian blue (AB-PAS) stains according to the manufacturers’ instructions. Images were acquired using a DMi8 microscope (Leica, Frankfurt, DE).

### 4.9. Transmission Electron Microscopy (TEM) Analysis

Colon tissue was fixed with 2.5% glutaraldehyde, sliced, and stained with 8% uranyl acetate and lead citrate. Images for TEM analysis were obtained using an HT7700 electron microscope (Hitachi, Tokyo, Japan).

### 4.10. Western Blotting (WB) Analysis

Colon tissue was pulverized in RIPA lysis buffer supplemented with protease and phosphatase inhibitors. Total protein was extracted, and protein content was quantified using the BCA protein assay kit (A55865, Thermo Scientific™, Waltham, MA, USA). Protein samples (30 µg) were separated by SDS-PAGE and transferred to PVDF membranes (Millipore, Burlington, MA, USA), followed by incubation with anti-ZO1 (21773-1-AP, 1:10,000, Proteintech, Wuhan, China), anti-Occludin (27260-1-AP, 1:10,000, Proteintech, Wuhan, China), anti-Claudin-2 (26912-1-AP, 1:1000, Proteintech, Wuhan, China), and anti-β-actin (bs-0061R, 1:5000, Bioss, Beijing, China) antibodies. Target protein bands were imaged and analyzed using an Amersham ImageQuant 2000 gel imaging system (Cytiva, Marlborough, CA, USA) and Image J software (v.2.3.0/1.53f), respectively.

### 4.11. Real-Time qPCR (RT-qPCR) Assay

Total RNA was extracted from colon tissues using an RNA extraction assay kit (Solarbio, Beijing, China) and reverse transcribed using a cDNA synthesis kit (Thermo Scientific™, K1691, Waltham, MA, USA). Quantitative PCR was employed to assess target gene expression, and the expression levels were normalized to GAPDH using the 2^−ΔΔCT^ method. Primer syntheses were performed by Sangon Biotech Co., Ltd. (Shanghai, China). The primer sequences are shown in Table 2.

### 4.12. Network Pharmacology Analysis

The determination of WLD components was conducted through a combined analysis of WLD and WLD drug-containing plasma mass spectrometry data. The corresponding targets of these components were retrieved from the TCM database (http://www.tcmsp-e.com/#/home, accessed on 12 April 2023) and the ETCM database (http://www.tcmip.cn/ETCM/, accessed on 12 April 2023). In addition, disease-related targets were obtained from the GeneCards database (http://www.genecards.org, accessed on 14 April 2023) and the Online Mendelian Inheritance in Man (OMIM) database (http://www.omim.org, accessed on 14 April 2023). Overlapping components and targets were identified using the jvenn online platform.

Protein–protein interaction (PPI) networks were constructed to explore interactions among proteins using the STRING database (https://string-db.org/, accessed on 14 April 2023). The networks were visualized using Cytoscape 3.7.2 software, while key targets within the PPI networks were identified using the CytoNCA plugin. Key targets were selected based on topological parameters, including degree centrality (DC), betweenness centrality (BC), and closeness centrality (CC), with thresholds set at values greater than or equal to the median. This approach enabled the identification of key targets relevant to WLD’s treatment of CDD. Gene Ontology (GO) terms and Kyoto Encyclopedia of Genes and Genomes (KEGG) pathway enrichment analyses were performed using the DAVID database (https://david.ncifcrf.gov/, accessed on 14 April 2023).

### 4.13. Molecular Docking

The three-dimensional structure of the target protein was retrieved from the PDB database to serve as the receptor. The mol2 formats of the seven quantitative components of WLD were obtained from the PubChem database to act as ligands. Molecular docking studies were performed using AutoDock Tools 1.5.7, while receptor–ligand interactions were visualized with PyMOL 2.4. The parameters for the molecular docking study were as follows: the center coordinate and grid size are provided in Table 3, the docking algorithm employed was AutoDock Vina, and the scoring was based on the criteria implemented in the AutoDock Vina software (v.1.5.7).

### 4.14. Serum Pharmacochemistry Trial

Rats were gavaged with a WLD dose of 2.16 g/kg (high-dose group) twice daily for 5 days. Blood samples were collected before and after the last treatment at various time points: 15 min, 30 min, 1 h, 2 h, 4 h, 8 h, 12 h, 24 h, 36 h, and 48 h. Plasma samples were stored at −80 °C for later analysis. To prepare for analysis, plasma samples were mixed with methanol and acetonitrile. After 30 s of vortexing, the mixture was centrifuged, and the supernatant was collected. This supernatant was freeze-dried and subsequently dissolved in methanol under sonication. After the precipitate was removed, the remaining supernatant was used for UPLC-MS/MS analysis (see the Supplementary Materials for specific analytical methods).

### 4.15. Isolation of Lymphocytes from the Mesenteric Lymph Nodes and Spleen

The lymph nodes were immersed in sterile PBS and cut into small pieces using surgical scissors. The pieces were then transferred to a 70 μm cell screen placed in a Petri dish containing 5 mL of PBS with 5% FBS. The tissue was ground with a syringe stopper until fully filtered. The resulting cell filtrate was resuspended in 15 mL of PBS with 5% FBS and centrifuged at 300 g for 5 min. After centrifugation, the cell pellet was collected and resuspended in a complete medium, adjusting the cell concentration to 10^7^ cells/mL for further experiments.

For spleen tissue, it was ground in PBS buffer to prepare a cell suspension. The cell suspension was then treated with a lysis solution (Cat. No. 420301, BioLegend, San Diego, CA, USA) to lyse the erythrocytes. Following this, the mixture was centrifuged at 300 g for 5 min, allowing the lymphocyte layer to be obtained. The lymphocyte cells were resuspended in 5 mL of PBS containing 5% FBS, and the suspension was centrifuged again at 300 g for 5 min to collect the cell pellet. The final concentration of the lymphocyte suspension was adjusted to 10^7^ cells/mL for subsequent use.

### 4.16. Flow Cytometry Analysis

Single-cell suspensions were used to assess Th1/Th2 and Th17/Treg cells. Subsequently, equal volumes of RPMI-1640 medium (Gibco, Thermo Fisher Scientific, MA, USA) were added to the samples, and a leukocyte activation cocktail was supplemented for cell stimulation, following the manufacturer’s instructions (423303, BioLegend, CA, USA). The stimulated lymphocytes were then labeled with CD4-APC-Cy7/CD25 AF488 and CD4-APC-Cy7 at 4 °C for 0.5 h. After washing, fixing, and permeabilizing the cells, staining was performed using Foxp3-APC and IL-17A FITC PE/IL-17A PE-Cy7 monoclonal antibodies at 4 °C for 0.5 h. All procedures were conducted in darkness. The final cell preparations were analyzed using flow cytometry (NL-CL3000, Cytek, Fremont, CA, USA) and FlowJo 10 software (Ashland, OR, USA).

### 4.17. Transcriptomic Sequencing

Total RNA was extracted from frozen colon tissues using the TRIzol^®^ Reagent kit (Invitrogen, Thermo Fisher Scientific, MA, USA). The concentration and purity of the extracted RNA were measured using a Nanodrop 2000 spectrophotometer (Thermo Fisher Scientific, MA, USA). RNA samples meeting the criteria of a concentration greater than 35 ng/μL, an OD260/280 ratio of 1.8~2.1, and a total RNA amount of at least 1 μg were selected for further analysis. These RNA samples were reverse transcribed to synthesize cDNA. Subsequently, the cDNA products were purified, fragmented, and amplified by PCR to construct the sequencing libraries. Adaptor ligation and additional PCR amplification were performed, and the final libraries were prepared for sequencing using the Illumina NovaSeq 6000 platform.

Bioinformatics analyses were conducted using the Majorbio online platform. Raw sequencing reads were processed with fastp software (v.0.23.4) for quality control, ensuring a read length of P150 and a sequencing depth exceeding 6 GB. Clean reads were assessed based on GC content and Q20 and Q30 scores and subsequently used for transcriptomics analysis. Differentially expressed genes (DEGs) were identified using DESeq2 software (v.1.23.10), with screening criteria set at |log2 (fold change)| ≥ 1 and adjusted *p* ≤ 0.05. Gene Ontology (GO) and Kyoto Encyclopedia of Genes and Genomes (KEGG) enrichment analyses were performed using GOatools (https://github.com/tanghaibao/GOatools, accessed on 22 May 2023) and the Python scipy package (https://scipy.org/install/, accessed on 22 May 2023). Significantly enriched terms or pathways were determined based on a corrected *p*-value ≤ 0.05.

### 4.18. Statistical Analysis

Data were expressed as mean ± standard deviation (SD). Statistical analyses were performed using GraphPad Prism version 9.3.1 to determine significant differences among groups. A Student’s *t*-test was employed between the two groups. One-way ANOVA followed by Tukey’s multiple comparisons test was utilized to assess differences among more than two groups. All *p*-values indicated significant differences at *p* < 0.05.

## 5. Conclusions

In this study, CDD was associated with an increase in inflammatory cytokines (MPO and IL-17), a disruption of gastrointestinal hormones (MTL and GAS), reduced levels of tight junction proteins (OCLN and ZO1), and an imbalance in the Th1/Th2 and Th17/Treg cell ratios. WLD administration effectively reduced intestinal inflammatory responses, restored gastrointestinal hormone levels, alleviated intestinal barrier damage, and promoted intestinal barrier function in rats with CDD. The potential mechanism underlying these effects involves the regulation of the Th1/Th2 and Th17/Treg cell balance, thereby restoring immune homeostasis and relieving CDD symptoms. This study elucidated the potential therapeutic mechanism of WLD in the treatment of CDD and provided valuable insights for further research on this condition. However, additional in vitro experiments are required to further validate these findings and explore their broader implications.

## Figures and Tables

**Table 1:** The criteria of CDD model scores.

Scores	Behavior Condition	Psychological Condition	Hair Condition	Fecal Condition	Dietary Condition	Weight
0	normal	normal	normal	normal	normal	increasing
1	tired and lazy	dispirited	fewer fall off	diarrhea	no increase	no weight gain
2	curled up in a pile	depressed state of mind	easy to fall off	Severe diarrhea	reduction	reduction

**Table 2:** Gene-specific primer sequences for RT-qPCR.

Genes	Forward (5′ to 3′)	Reverse (5′ to 3′)	Production Length (bp)	GenBank Accession No.
T-bet	CTACTCACCTCTTCTGTCGAAC	GCTCGGAACTCTGTTTCATAAC	141	NM_001107043.1
IL-21	AACTTCTAACAGCTCCACAAGA	GTGCCTCTGTTTATTTCCTGTC	179	NM_001108943.3
IL-23	ACAACAGCTCGAGTTTGGTATA	AATGAGTGTCTCTTGAAAACGC	96	XM_039108697.1
IL-17a	CTGTTGCTGCTACTGAACCTGGAG	CCTCGGCGTTTGGACACACTG	82	NM_001106897.1
IL-17f	CGTCTCTTTGCGTTAGATGATG	GCACTTCATTGAGCTCTACAAG	91	NM_001015011.2
RORγt	GCAAGTTCAACGGCACAG	GCCAGTAGACTCCACGACAT	140	NM_017008.4
RORa	CAATATACCCAGACATTGTGCG	ACTCCAGATGTTCTAGAAGTGC	130	XM_008766410.3
STAT4	TCAAGAAAGAACAGCCCATTTG	TGAAGTCCTTCAGAGTAACAGG	87	NM_032612.3
STAT6	GTCTTGGTCACAGTTCAACAAG	TGCTTACTGATAAAGCCGATGA	144	NM_001044250.1
GATA3	GGATCCCATTTGTGAATAAGCC	CCCTTAAAATTCTTGGCGTCTT	194	NM_133293.2
TGF-β1	CTTCAATACGTCAGACATTCGG	CACAGTTGACTTGAATCTCTGC	91	NM_021578.2
Foxp3	TCACACGCATGTTCGCCTACTTC	CTCACTCTCCACTCGCACAAAGC	104	NM_001108250.1
Smad3	CATGTGGCTTATAGTCATGTGC	CTCTAGGCTTTCCTCAAAATGC	95	NM_013095.3
GAPDH	GCAAGTTCAACGGCACAG	GCCAGTAGACTCCACGACAT	140	NM_017008.4

**Table 3:** Docking parameters of the target proteins.

Target Proteins	PDB ID	Center Coordinate (x, y, z)/mn	Grid Size (x × y × z)/mn
Cd3d	1XIW	45.958, −10.552, 14.348	38.0 × 34.0 × 40.0
Cd247	6GXR	129.43, 142.682, 150.256	48.0 × 26.0 × 42.0
IL2ra	1Z92	14.048, −33.637, 12.454	40.0 × 44.0 × 54.0
IL2rb	2B5I	−14.048, −40.577, 20. 537	58.0 × 40.0 × 78.0
IL27ra	7U7N	99.085, 126.994, 96.003	80.0 × 46.0 × 40.0
Irf4	5BVI	−23.981, 11.496, 23. 694	40.0 × 48.0 × 58.0
Lat	4XGC	−5.169, −48.462, 73.098	72.0 × 96.0 × 96.0
Lck	1X27	49.351, 40.485, 119.931	64.0 × 40.0 × 46.0
Nfatc2	1S9K	19.537, 20.574, 70.453	60.0 × 56.0 × 58.0
Tgfbr1	6MAC	48.797, 0.042, 13.68	40.0 × 44.0 × 40.0

## Data Availability

All data that support the findings of this study are available upon request from the corresponding author.

## Supplementary Materials

The following supporting information can be downloaded at: https://www.mdpi.com/article/10.3390/ph18010109/s1. Figure S1: Quantitative analysis of WLD and its representative compounds by UPLC-MS/MS. Figure S2: The standard curve of standards used in UPLC-MS/MS. Figure S3: The establishment of the model and evaluation of CDD. Figure S4: Transcriptomics analysis of Th1/Th2 and Th17/Treg cell-associated differentiation genes among the control, model, and WLD groups. Table S1: Regression equations, correlation coefficients, and linear ranges of the seven components. Table S2: Chemical components of WLD by UPLC-MS/MS. Table S3: Entry-blood components of WLD by UPLC-MS/MS. Table S4: The specific herb information of WLD. Table S5: The main symptoms of CDD model.
