# Supplementary material for: Exploring the Underlying Mechanism of Weiling Decoction Alleviates Cold-Dampness Diarrhea Based on Network Pharmacology, Transcriptomics, Molecular Docking and Experimental Validation
Source: Pharmaceuticals (Basel). 2025 Jan 16;18(1):109. doi: 10.3390/ph18010109 (PMC11768181; doi:10.3390/ph18010109)
Supplement: Supplementary file 1 [file pharmaceuticals-18-00109-s001.zip › pharmaceuticals-3354971-supplementary.pdf]

Supplementary material information

1. Methods

1.1 Detection methods in main chemical components analysis for WLD by UHPLC-MS/MS

This experiment was a UHPLC-MS/MS system performed with Q Exactive Orbitrap (Thermo Fisher Scientific, USA), a high-resolution tandem mass spectrometer with an iron source. The chromatographic separation was performed on an ACQUITY UPLC HSS T3 column (2.1 × 100 mm, 1.8 μm, Waters) at a flow rate of 0.3 ml/min with the column temperature kept at 35 °C. The mobile phase was 0.1% formic acid (A)-acetonitrile (B).

Table MS1 The elution produce

Time (min)	A	B
0	100%	0%
10	70%	30%
25	60%	40%
30	50%	50%
40	10%	90%
45	0%	100%
60	0%	100%
60.5	100%	0%
70	100%	0%

The mass spectral analysis was obtained in full MS-ddMS^2 mode and full scan analysis. The parameters are as follows: scan range, m/z 100-1200; MS^1 and MS^2 resolution, 70,000 and 17,500. The data were performed in both positive and negative ionization modes for qualitative analysis of the samples. Compound Discoverer 3.3 software was employed to acquire raw data, and both the local and online MzCloud databases were simultaneously utilized to identify compounds.

1.2 Detection methods in main chemical components analysis for WLD by UHPLC-MS/MS

The analytes were detected using an QTRAP5500 tandem quadrupole mass spectrometer (ABSCIEX，USA) with an electrospray ionization (ESI) interface in positive ion mode and negative mode. Multiple reaction monitoring (MRM) was used to monitor the precursor to product ion transition. The source parameters were ion spray voltage, 5500~4500V; turbo heater temperature, 500°C; collision activation dissociation, medium; Misting gas and auxiliary heating gas, 50 psi and curtain gas, 25 psi. The analytical data was processed using Analyst software (version 1.6.1, Applied ABSCIEX OS).

1.3 Detection methods for serum pharmacochemistry

A chromatographic separation was conducted using a Waters ACQUITY UPLC HSS T3 C18 column (2.1 mm × 100 mm, 1.8 μm; Waters Corporation, USA). The column temperature was set at 35 °C, and the flow rate was maintained at 0.2 mL/min. The mobile phase consisted of 0.1% formic acid in acetonitrile (solution A) and 0.1% formic acid in water (solution B). Both positive and negative ionization modes of the mass spectrometer were employed for analysis. The ion discharge voltages were set to 3.2 kV for positive mode and 3.0 kV for negative mode. The scan mass ratio for the positive ion mode ranged from 100 to 1500 m/z. Data analysis was performed using the Compound Discoverer 3.3, mzCloud, and mzVault databases.

2. Results

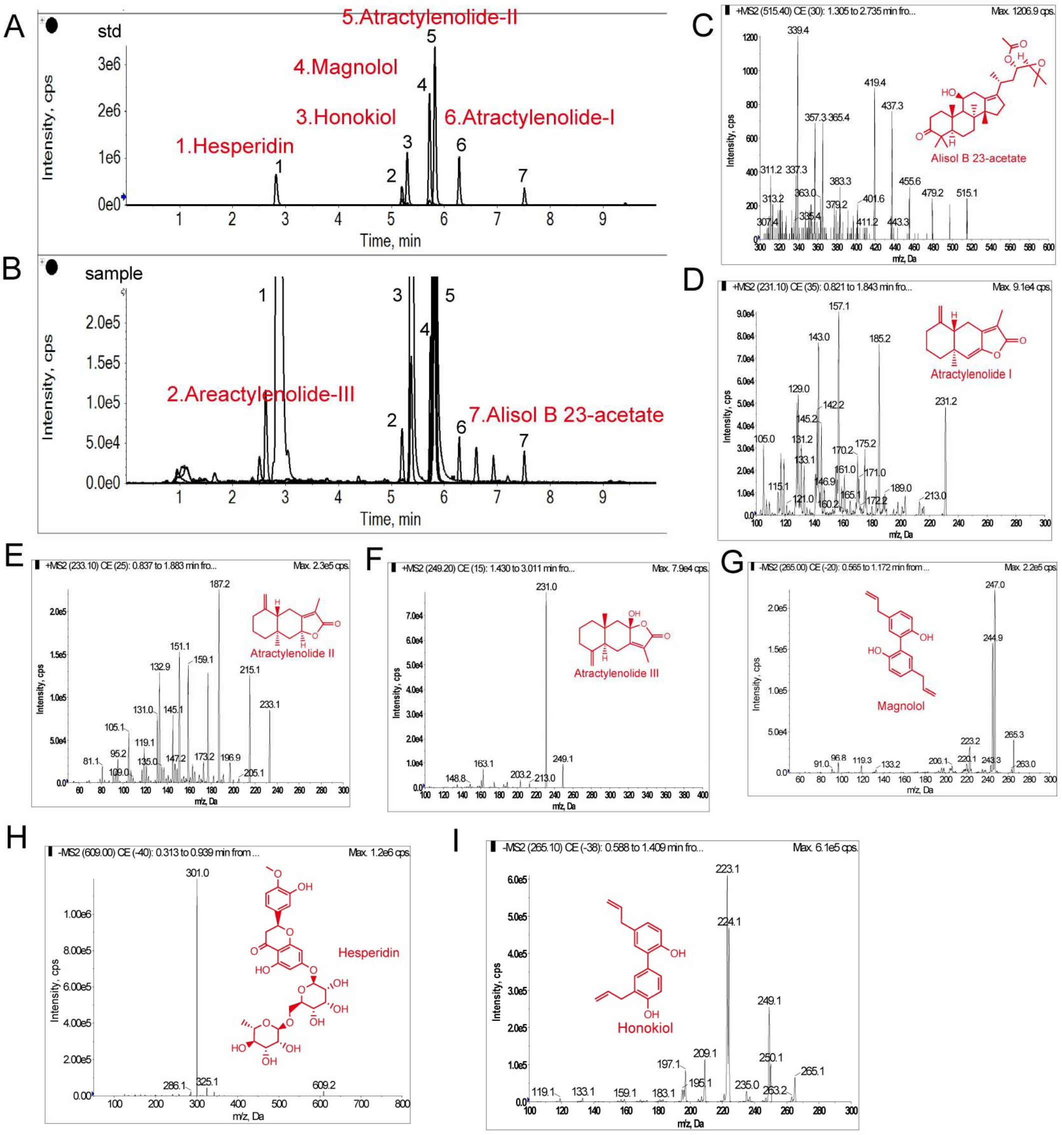

Figure S1. Quantitative analysis of WLD and its representative compounds by UPLC-MS/MS. (A) Total ion flow diagram of the standard products; (B) Total ion flow diagram of the sample.

(C-I) The secondary mass spectrometry diagram of Alisol B 23-acetate, Atractylenolide-I, Atractylenolide-II, Atractylenolide-III, Magnolol, Hesperidin, Honokiol.

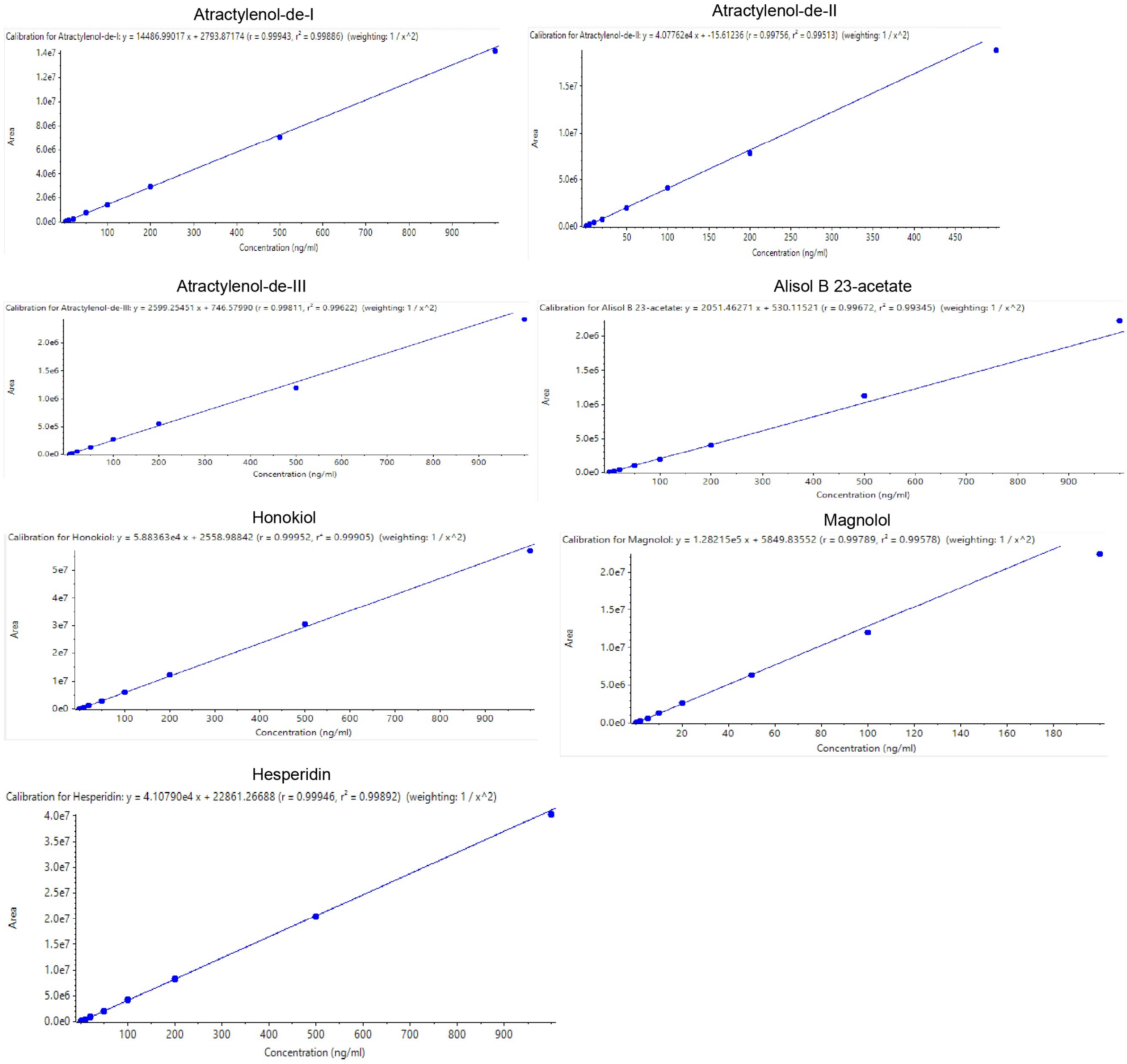

Figure S2. The standard curve of standards used in UPLC-MS/MS.

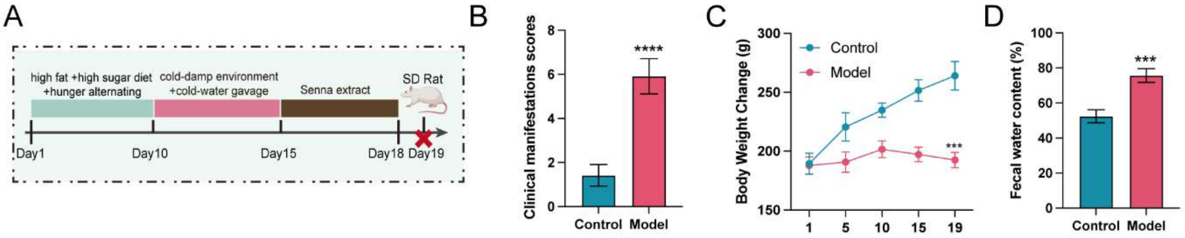

Figure S3. The establishment of the model and evaluation of CDD. (**A**) The process of model building for CDD. (**B**) Clinical symptom score for the CDD model, n=6. (**C**) Weight change during model building, n=6. (**D**) Changes in the fecal water content after model establishment, n=6.

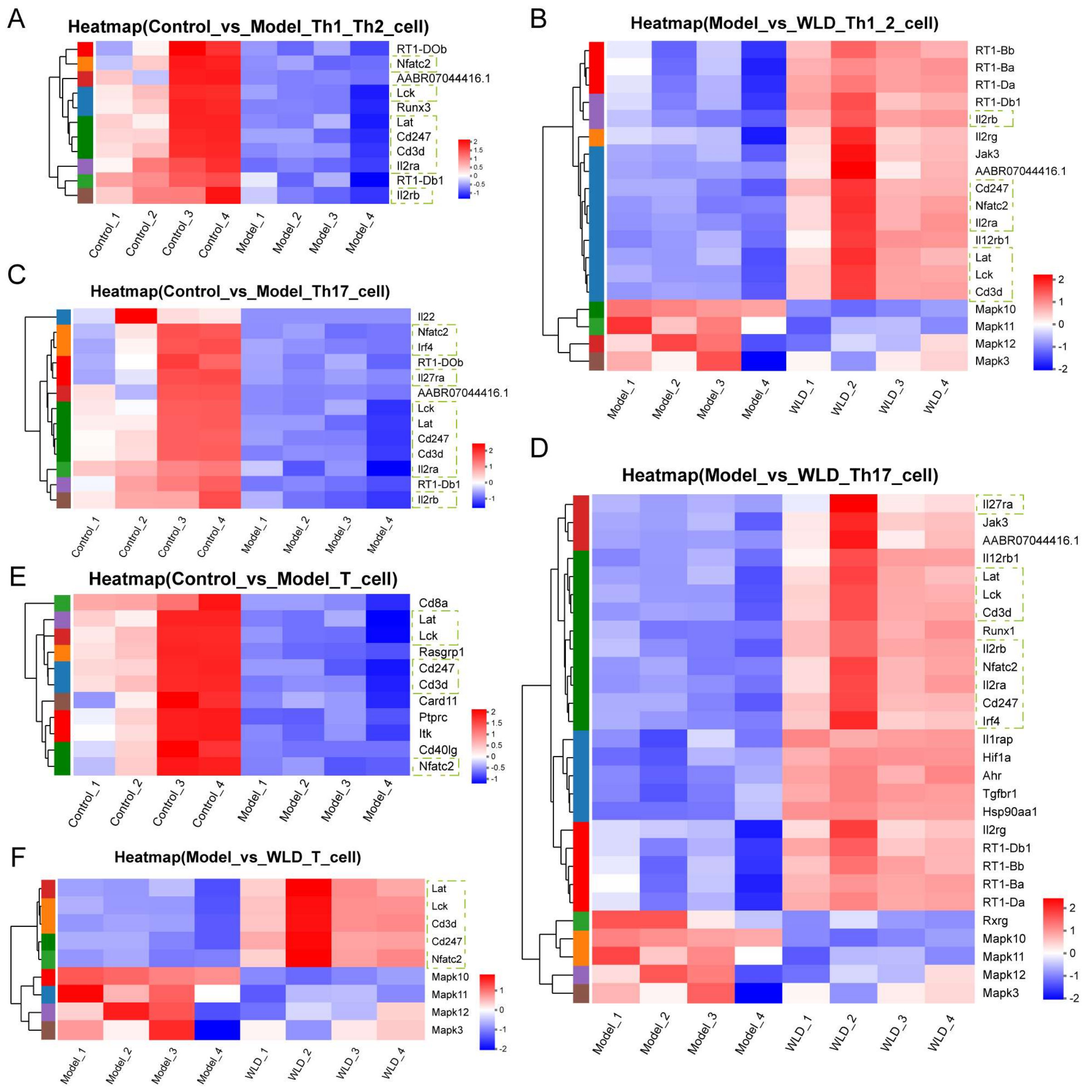

Figure S4. Transcriptomics analysis of Th1/Th2 and Th17/Treg cell-associated differentiation genes among the control, model, and WLD groups. (**A** and **B**) Th1 and Th2 cell differentiation associated differentiation genes among three groups. n=4. (**C** and **D**) Th17 cell differentiation associated differentiation genes among three groups. n=4. (**E** and **F**) T cell receptor associated differentiation genes among control, model, and WLD group. n=4.

Table S1. Regression equations, correlation coefficients, and linear ranges of the seven components.

Analytes	Linearity equation	R2	Linear range(ng/g)	WLD (μg/g, mean ± SD)
Atractylenolide I	y = 1.45 × 10^4 X+2793.87	0.99943	0.2~1000	9.38 ± 4.63
Atractylenolide II	y = 4.08 × 10^4 X-15.61	0.99513	0.5~500	30.71 ± 7.60
Atractylenolide III	y = 2.60 × 10^3 X + 746.58	0.99622	5~1000	100.03 ± 3.77

Alisol B 23-acetate	y = 2.05 × 10^3 X + 530.12	0.99345	1~1000	28.24 ± 7.54
Honokiol	y = 5.88 × 10^4 X + 2558.99	0.99905	0.05~1000	420.1 ± 13.63
Magnolol	y = 1.28 × 10^5 X + 5849.84	0.99578	0.05~200	513.68 ± 104.47
Hesperidin	y = 4.11 × 10^4 X + 22861.27	0.99892	0.05~1000	5.23 ± 0.62

Table S2 Chemical components of WLD by UPLC-MS/MS.

No.	Name	Formula	Calc. MW	RT [min]	Reference Ion	Group Area	Classification
1	Hesperidin	C28 H34 O15	610.18522	25.864	[M-H]-1	5.28E+09	flavonoids
2	α,α-Trehalose	C12 H22 O11	342.11452	1.626	[M-H]-1	4.55E+09	Glycosides
3	Magnolol	C18 H18 O2	266.12883	41.675	[M-H]-1	3.46E+09	phenylpropanoids
4	(+)-Magnoflorine	C20 H23 N O4	341.15999	23.431	[M+H]+1	2.70E+09	Alkaloids
5	Narirutin	C27 H32 O14	580.17549	25.263	[M-H]-1	2.50E+09	flavonoids
6	N-(2,4-Dimethylphenyl)formamide	C9 H11 N O	149.08342	1.709	[M+H]+1	2.25E+09	Organic acids
7	DL-Stachydrine	C7 H13 N O2	143.09398	1.625	[M+H]+1	2.24E+09	Alkaloids
8	L-(-)-Malic acid	C4 H6 O5	134.0209	1.715	[M-H]-1	2.18E+09	Organic acids
9	Cathinone	C9 H11 N O	149.08342	3.139	[M+H]+1	2.04E+09	Flavonoids
10	Choline	C5 H13 N O	103.09912	1.43	[M+H]+1	1.95E+09	Miscellaneous
11	Gluconic acid	C6 H12 O7	196.0574	1.491	[M-H]-1	1.86E+09	Glycosides
12	L-Pyroglutamic acid	C5 H7 N O3	129.04194	1.446	[2M+H]+1	1.73E+09	Amino acids
13	D-(-)-Quinic acid	C7 H12 O6	192.06257	1.6	[M-H]-1	1.55E+09	Organic acids
14	Sucrose	C12 H22 O11	342.11452	1.972	[M-H]-1	1.54E+09	Glycosides
15	Isoforsythiaside	C29 H36 O15	624.20133	23.505	[M-H]-1	1.53E+09	Phenylpropanoids
16	Hesperetin	C16 H14 O6	302.07623	25.855	[M+H]+1	1.19E+09	Flavonoids
17	Nobiletin	C21 H22 O8	402.12825	35.638	[M+H]+1	1.03E+09	Flavonoids
18	Diammonium glycyrrhizinate	C42 H62 O16	822.39801	33.17	[M-H]-1	9.07E+08	Organic acids
19	2-Furoic acid	C5 H4 O3	112.01548	3.545	[M-H]-1	7.36E+08	Organic acids
20	D-Raffinose	C18 H32 O16	504.16676	1.62	[M+FA-H]-1	7.16E+08	Glycosides
21	Purpureaside C	C35 H46 O20	786.25314	22.638	[M-H]-1	6.00E+08	Glycosides
22	Tangeritin	C20 H20 O7	372.11805	37.427	[M+H]+1	5.53E+08	Flavonoids
23	Liquiritin	C21 H22 O9	418.12376	24.812	[M-H]-1	5.51E+08	Flavonoids
24	5-Hydroxymethyl-2-furaldehyde	C6 H6 O3	126.03111	1.549	[M+H]+1	5.50E+08	Glycosides
25	D-(-)-Fructose	C6 H12 O6	180.06264	1.536	[M-H]-1	5.47E+08	Glycosides
26	Liguiritigenin-7-O-β-D-apiosyl-4'-O-β-D-glucoside	C26 H30 O13	550.16515	24.577	[M-H]-1	5.43E+08	Glycosides
27	6-Gingerol	C17 H26 O4	276.17034	35.617	[M+H]+1	5.14E+08	Flavonoids
28	Isoliquiritigenin	C15 H12 O4	256.07137	24.818	[M+H]+1	5.12E+08	Flavonoids
29	Chlorogenic acid	C16 H18 O9	354.09277	21.662	[M-H]-1	4.61E+08	Phenylpropanoids
30	Stachyose	C24 H42 O21	666.21911	1.632	[M-H]-1	4.31E+08	Glycosides
31	Pipecolic acid	C6 H11 N O2	129.07842	1.67	[M+H]+1	4.31E+08	Organic acids
32	L-Aspartic acid	C4 H7 N O4	133.03687	1.395	[M-H]-1	4.27E+08	Amino acids
33	Naringenin	C15 H12 O5	272.06608	25.276	[M+H]+1	4.01E+08	Flavonoids
34	L-Phenylalanine	C9 H11 N O2	165.0779	12.895	[M+H]+1	3.20E+08	Amino acids
35	Erucamide	C22 H43 N O	337.33174	52.223	[M+H]+1	3.17E+08	Organic acids
36	7-Hydroxycoumarine	C9 H6 O3	162.03037	22.626	[M+H]+1	3.00E+08	Flavonoids
37	Betaine	C5 H11 N O2	117.07838	1.514	[M+H]+1	3.00E+08	Alkaloids
38	Trigonelline	C7 H7 N O2	137.04701	1.62	[M+H]+1	2.98E+08	Alkaloids
39	(15Z)-9,12,13-Trihydroxy-15-octadecenoic acid	C18 H34 O5	330.23849	31.802	[M-H]-1	2.93E+08	Organic acids

40	Didymin	C28 H34 O14	594.1906	28.332	[M+FA-H]-1	2.85E+08	Flavonoids
41	Leucine	C6 H13 N O2	131.0939 7	5.029	[M+H]+1	2.22E+08	Amino acids
42	Fumaric acid	C4 H4 O4	116.0103 4	1.72	[M-H]-1	2.22E+08	Phenols
43	3-(4-Hydroxyphenyl)propionic acid	C9 H10 O3	120.0564 3	22.287	[M+FA-H]-1	2.18E+08	Organic acids
44	β-D-Glucopyranuronic acid	C6 H10 O7	194.0417 8	1.495	[M-H]-1	2.14E+08	Glycosides
45	Oleamide	C18 H35 N O	281.2699 3	48.239	[M+H]+1	1.88E+08	Organic acids
46	Forsythoside E	C20 H30 O12	462.1710 1	21.551	[M-H]-1	1.82E+08	Phenylpropanoids
47	Dehydrocostus lactone	C15 H18 O2	230.1288 6	37	[M+H]+1	1.77E+08	Flavonoids
48	Skimmin	C15 H16 O8	324.0818 4	23.508	[M+H]+1	1.73E+08	Flavonoids
49	Mangostin	C24 H26 O6	410.1701 1	46.499	[M+H]+1	1.71E+08	Flavonoids
50	Liquiritigenin	C15 H12 O4	256.0716 3	28.627	[M-H]-1	1.69E+08	Flavonoids
51	Plantamajoside	C29 H36 O16	640.1966 1	22.244	[M-H]-1	1.64E+08	Phenylpropanoids
52	Agmatine	C5 H14 N4	130.1212 5	1.258	[M+H]+1	1.64E+08	Amino acids
53	6-Demethoxytangeretin	C19 H18 O6	342.1075 5	34.083	[M+H]+1	1.59E+08	Flavonoids
54	Honokiol	C18 H18 O2	266.1288 7	39.725	[M-H]-1	1.58E+08	Phenols
55	trans-Aconitic acid	C6 H6 O6	174.0155 9	3.559	[M+H]+1	1.57E+08	Organic acids
56	Adenine	C5 H5 N5	135.0539 2	2.811	[M+H]+1	1.54E+08	Miscellaneous
57	3-Methoxybenzaldehyde	C8 H8 O2	136.0513 9	35.618	[M+H]+1	1.52E+08	Phenols
58	Sinensetin	C20 H20 O7	372.1178 7	34.01	[M+H]+1	1.49E+08	Flavonoids
59	Atractylenolide II	C15 H20 O2	232.1445 5	41.069	[M+H]+1	1.43E+08	Flavonoids
60	2,5-Dihydroxybenzaldehyde	C7 H6 O3	138.0308 4	21.967	[M-H]-1	1.41E+08	Phenols
61	Isosinensetin	C20 H20 O7	372.1178 2	32.452	[M+H]+1	1.38E+08	Flavonoids
62	Rutin	C27 H30 O16	610.1492 9	24.393	[M-H]-1	1.34E+08	Flavonoids
63	Prunin	C21 H22 O10	434.1177 2	25.266	[M+H]+1	1.27E+08	Flavonoids
64	Ononin	C22 H22 O9	430.1227 5	27.284	[M+H]+1	1.20E+08	Flavonoids
65	7-Methoxycoumarin	C10 H8 O3	176.0460 8	23.035	[M+H]+1	1.18E+08	Flavonoids
66	Synephrine	C9 H13 N O2	167.0938 6	1.708	[M+H]+1	1.16E+08	Phenols
67	Nicotinamide	C6 H6 N2 O	122.0474	3.324	[M+H]+1	1.08E+08	Organic acids
68	Glabrolide	C30 H44 O4	468.3205 4	30.743	[M+H]+1	1.07E+08	Flavonoids
69	3-Hydroxy-3-(methoxycarbonyl)pentanedioic acid	C7 H10 O7	206.0414 7	12.834	[M-H]-1	1.03E+08	Organic acids
70	Isosakuranetin	C16 H14 O5	286.0815 8	28.325	[M+H]+1	1.02E+08	Flavonoids
71	BMK methyl glycidate	C11 H12 O3	192.0772 6	22.39	[M+H]+1	9.74E+07	Miscellaneous
72	trans-3-Indoleacrylic acid	C11 H9 N O2	187.0619 2	21.675	[M+H]+1	9.59E+07	Organic acids
73	α-D-Mannose 1-phosphate	C6 H13 O9 P	260.0284 6	1.436	[M-H]-1	9.38E+07	Miscellaneous
74	Eriocitrin	C27 H32 O15	596.1704 8	24.29	[M-H]-1	9.22E+07	Flavonoids
75	Isoliquiritin	C21 H22 O9	418.1235 2	27.21	[M-H]-1	8.83E+07	Flavonoids
76	Nicotinic acid	C6 H5 N O2	123.0315	2.867	[M+H]+1	8.07E+07	Organic acids
77	1-Naphthol	C10 H8 O	144.0564 1	35.618	[M+H]+1	8.07E+07	Phenols
78	Atractylenolide I	C15 H18 O2	230.1288 6	43.771	[M+H]+1	7.60E+07	Flavonoids
79	Alisol C 23-acetate	C32 H48 O6	528.3412 7	40.105	[M+H]+1	7.54E+07	Terpenoids
80	Ferulaldehyde	C10 H10 O3	178.0615 9	35.618	[M+H]+1	7.52E+07	Miscellaneous
81	Formononetin	C16 H12 O4	268.0713 7	33.101	[M+H]+1	7.00E+07	Flavonoids
82	Melittoside	C21 H32 O15	524.1707 3	22.442	[M-H]-1	6.87E+07	Glycosides
83	Shogaol	C17 H24 O3	276.1703 4	41.33	[M+H]+1	6.72E+07	Phenols
84	DL-Tryptophan	C11 H12 N2 O2	204.0884 1	21.663	[M+H]+1	6.54E+07	Amino acids
85	Guanosine	C10 H13 N5 O5	283.0901 9	13.817	[M-H]-1	6.50E+07	Nucleotides

86	L-Tyrosine	C9 H11 N O3	181.07302	5.474	[M+H]+1	6.11E+07	Amino acids
87	18 β-Glycyrrhetintic Acid	C30 H46 O4	470.33592	33.184	[M+H]+1	5.59E+07	Terpenoids
88	Limonin	C26 H30 O8	470.19054	24.715	[M+H]+1	5.54E+07	Terpenoids
89	Atractylenolide III	C15 H20 O3	248.13921	36.999	[M+H]+1	5.53E+07	Phenylpropanoids
90	Amygdalin	C20 H27 N O11	457.15544	22.786	[M+FA-H]-1	5.32E+07	Miscellaneous
91	Alisol B 23-acetate	C32 H50 O5	514.36217	47.558	[M+H]+1	5.25E+07	Terpenoids
92	Rhoifolin	C27 H30 O14	578.15988	25.326	[M+FA-H]-1	5.24E+07	Flavonoids
93	Succinic acid	C4 H6 O4	118.02605	4.484	[M-H]-1	5.14E+07	Organic acids
94	Azelaic acid	C9 H16 O4	188.1039	26.559	[M-H]-1	5.06E+07	Organic acids
95	Caffeic acid	C9 H8 O4	180.04112	23.207	[M-H]-1	4.84E+07	Organic acids
96	Isoferulic acid	C10 H10 O4	194.05652	23.031	[M+H]+1	4.78E+07	Organic acids
97	Icaritin	C21 H20 O6	368.12336	36.372	[M-H]-1	4.63E+07	Flavonoids
98	4-Coumaric acid	C9 H8 O3	164.04626	24.669	[M-H]-1	4.62E+07	Lactones
99	Argininosuccinic acid	C10 H18 N4 O6	290.12114	3.075	[M+H]+1	4.49E+07	Organic acids
100	Ferulic acid	C10 H10 O4	194.05652	22.556	[M+H]+1	4.32E+07	Organic acids
101	Ibuprofen	C13 H18 O2	206.12912	23.233	[M+H]+1	4.29E+07	Organic acids
102	4-Guanidinobutyric acid	C5 H11 N3 O2	145.0844	2.798	[M+H]+1	4.29E+07	Organic acids
103	4-Vinylphenol	C8 H8 O	120.05665	24.66	[M-H]-1	4.20E+07	Phenols
104	Acetylarginine	C8 H16 N4 O3	216.12135	2.954	[M+H]+1	4.08E+07	Organic acids
105	Uridine	C9 H12 N2 O6	244.06831	5.772	[M-H]-1	3.98E+07	Miscellaneous
106	Poliumoside	C35 H46 O19	770.2588	23.136	[M-H]-1	3.96E+07	Phenylpropanoids
107	Calceolarioside B	C23 H26 O11	478.14464	23.901	[M-H]-1	3.78E+07	Miscellaneous
108	Corchorifatty acid F	C18 H32 O5	328.22285	30.706	[M-H]-1	3.71E+07	Organic acids
109	Lonicerin	C27 H30 O15	594.1546	24.436	[M-H]-1	3.43E+07	Flavonoids
110	Bioside	C20 H30 O12	462.17096	22.306	[M-H]-1	3.41E+07	Glycosides
111	Isosakuranin	C22 H24 O10	448.13304	28.323	[M+H]+1	3.38E+07	Flavonoids
112	Lariciresinol 4-O-glucoside	C26 H34 O11	522.2068	24.574	[M+FA-H]-1	3.29E+07	Lignans
113	Wilforlide A	C30 H46 O3	454.34133	41.931	[M+H]+1	3.21E+07	Terpenoids
114	trans-Cinnamaldehyde	C9 H8 O	132.05654	22.401	[M+H]+1	3.14E+07	Terpenoids
115	2,3-Dihydroxybenzoic acid	C7 H6 O4	154.02569	18.462	[M-H]-1	3.07E+07	Organic acids
116	2''-O-β-L-Galactopyranosylorientin	C27 H30 O16	610.14948	22.498	[M-H]-1	2.99E+07	Glycosides
117	4-Hydroxy-3-methoxymethamphetamine (HMMA)	C11 H17 N O2	195.12491	5.549	[M+H]+1	2.98E+07	Miscellaneous
118	Cytosine	C4 H5 N3 O	111.04269	2.914	[M+H]+1	2.89E+07	Miscellaneous
119	Vicenin III	C26 H28 O14	564.14389	23.518	[M+H]+1	2.86E+07	Flavonoids
120	Boldine	C19 H21 N O4	327.14433	23.916	[M+H]+1	2.80E+07	Alkaloids
121	N-Acetylalanine	C5 H9 N O3	131.05745	1.456	[M+FA-H]-1	2.65E+07	Amino acids
122	Methyl cinnamate	C10 H10 O2	162.0668	34.606	[M+H]+1	2.64E+07	Lactones
123	N-Acetyl-L-phenylalanine	C11 H13 N O3	207.08816	24.503	[M-H]-1	2.62E+07	Amino acids
124	Isorhamnetin-3-O-nehesperidine	C28 H32 O16	624.16427	25.313	[M+H]+1	2.52E+07	Flavonoids
125	Clareolide	C16 H26 O2	250.19141	41.65	[M+H]+1	2.49E+07	Terpenoids
126	Benzoic acid	C7 H6 O2	122.03594	23.596	[M-H]-1	2.49E+07	Organic acids
127	2-Naphthylamine	C10 H9 N	143.07242	22.645	[M+H]+1	2.38E+07	Miscellaneous
128	Quercetin	C15 H10 O7	302.04021	24.383	[M+H]+1	2.32E+07	Flavonoids
129	9-Oxo-ODE	C18 H30 O3	294.21715	31.81	[M+H]+1	2.29E+07	Organic acids
130	Cinnamic acid	C9 H8 O2	148.05118	22.221	[M+H]+1	2.29E+07	Organic acids
131	(+/-)12(13)-DiHOME	C18 H34 O4	314.24358	38.648	[M-H]-1	2.22E+07	Miscellaneous

132	4-Methoxybenzaldehyde	C8 H8 O2	136.05139	34.645	[M+H]+1	2.22E+07	Terpenoids
133	2-Methylbenzoic acid	C8 H8 O2	136.05157	23.205	[M-H]-1	2.20E+07	Organic acids
134	(+/-)9,10-dihydroxy-12Z-octadecenoic acid	C18 H34 O4	314.2436	39.026	[M-H]-1	2.18E+07	Organic acids
135	Parathion-oxon	C10 H14 N O6 P	275.05603	27.915	[M+H]+1	2.09E+07	Terpenoids
136	(-)-Caryophyllene oxide	C15 H24 O	220.18085	33.25	[M+H]+1	2.04E+07	Terpenoids
137	α-Cyperone	C15 H22 O	218.16547	39.168	[M+H]+1	2.02E+07	Terpenoids
138	4-Indolecarbaldehyde	C9 H7 N O	145.05167	21.674	[M+H]+1	2.02E+07	Miscellaneous
139	3,5-Dicaffeoylquinic acid	C25 H24 O12	516.12354	25.663	[M-H]-1	2.01E+07	Organic acids
140	6-Methylquinoline	C10 H9 N	143.07242	21.675	[M+H]+1	1.96E+07	Organic acids
141	Scopoletin	C10 H8 O4	192.04082	25.323	[M+H]+1	1.94E+07	Alkaloids
142	Ursonic acid	C30 H46 O3	454.34126	45.557	[M+H]+1	1.83E+07	Organic acids
143	Kaempferol-3-O-rutinoside	C27 H30 O15	594.15463	25.205	[M-H]-1	1.82E+07	Flavonoids
144	Abscisic acid	C15 H20 O4	264.13409	28.504	[M-H]-1	1.80E+07	Terpenoids
145	Apigenin 7-O-(2G-rhamnosyl)gentiobioside	C33 H40 O19	740.2107	23.125	[M+H]+1	1.77E+07	Flavonoids
146	Salsolinol	C10 H13 N O2	179.09374	5.719	[M+H]+1	1.77E+07	Organic acids
147	4',7-Dihydroxyflavanone	C15 H12 O4	256.07134	27.205	[M+H]+1	1.69E+07	Flavonoids
148	2-Hydroxy-4-methoxybenzaldehyde	C8 H8 O3	152.04622	24.687	[M+H]+1	1.68E+07	Miscellaneous
149	5-O-Demethylnobiletin	C20 H20 O8	388.11278	39.057	[M+H]+1	1.67E+07	Flavonoids
150	Eriodictyol	C15 H12 O6	288.06099	24.28	[M+H]+1	1.61E+07	Flavonoids
151	5,7,3',4'-Tetrahydroxy-6,8-diprenylisoflavone	C25 H26 O6	422.17004	43.786	[M+H]+1	1.61E+07	Flavonoids
152	Coumarin	C9 H6 O2	146.03569	24.665	[M+H]+1	1.60E+07	Miscellaneous
153	2-Amino-3-methoxybenzoic acid	C8 H9 N O3	167.05751	3.419	[M+H]+1	1.60E+07	Miscellaneous
154	Cryptochlorogenic acid	C16 H18 O9	354.09269	21.233	[M-H]-1	1.49E+07	Organic acids
155	Scoparone	C11 H10 O4	206.05631	27.713	[M+H]+1	1.48E+07	Lactones
156	γ-mangostin	C23 H24 O6	396.15436	45.092	[M+H]+1	1.45E+07	Flavonoids
157	2'-O-Methyladenosine	C11 H15 N5 O4	281.11058	20.656	[M+H]+1	1.43E+07	Miscellaneous
158	Quillaic acid	C30 H46 O5	486.33087	33.037	[M+H]+1	1.43E+07	Terpenoids
159	Demethylcoclaurine hydrochloride	C16 H17 N O3	271.11876	21.907	[M+H]+1	1.41E+07	Alkaloids
160	4-Hydroxybenzoic acid	C7 H6 O3	138.03084	27.108	[M-H]-1	1.37E+07	Organic acids
161	α-Linolenic acid	C18 H30 O2	278.2224	47.169	[M+H]+1	1.35E+07	Organic acids
162	Arteannuin	C15 H20 O3	248.13921	30.163	[M+H]+1	1.34E+07	Lactones
163	Quinic acid	C7 H12 O6	192.06215	21.654	[M-H]-1	1.33E+07	Organic acids
164	Uridine monophosphate (UMP)	C9 H13 N2 O9 P	324.03435	2.531	[M-H]-1	1.32E+07	Miscellaneous
165	Padmatin	C16 H14 O7	318.07152	23.279	[M+H]+1	1.27E+07	Flavonoids
166	(3β,5ξ,9ξ)-3,23-Dihydroxy-1-oxoolean-12-en-28-oic acid	C30 H46 O5	486.33089	32.099	[M+H]+1	1.27E+07	Organic acids
167	Isokaempferide	C16 H12 O6	300.06126	26.28	[M+H]+1	1.26E+07	Flavonoids
168	Curcumol	C15 H24 O2	236.17576	37.856	[M+H]+1	1.25E+07	Flavonoids
169	3-tert-Butyladipic acid	C10 H18 O4	202.11929	28.649	[M-H]-1	1.24E+07	Organic acids
170	Parthenolide	C15 H20 O3	248.13921	30.494	[M+H]+1	1.24E+07	Terpenoids
171	3,5-Dimethoxy-4-hydroxybenzaldehyde	C9 H10 O4	182.05654	25.072	[M+H]+1	1.23E+07	Miscellaneous
172	Retrochalcone	C16 H14 O4	270.08719	30.767	[M+H]+1	1.17E+07	Flavonoids
173	N-Acetylglutamic acid	C7 H11 N O5	189.06294	4.481	[M-H]-1	1.16E+07	Miscellaneous
174	Diosmin	C28 H32 O15	608.17061	25.761	[M-H]-1	1.15E+07	Flavonoids
175	16-Hydroxyhexadecanoic acid	C16 H32 O3	272.23332	47.184	[M-H]-1	1.14E+07	Organic acids
176	Glabrone	C20 H16 O5	336.09729	41.679	[M+H]+1	1.14E+07	Flavonoids
177	Daidzein	C15 H10 O4	254.05597	27.82	[M+H]+1	1.13E+07	Flavonoids

178	4,5-Dicaffeoylquinic acid	C25 H24 O12	516.12356	26.161	[M-H]-1	1.12E+07	Phenols
179	8-Hydroxy-6-methoxy-2-oxo-2H-chromen-7-yl β-D-glucopyranoside	C16 H18 O10	370.0876	22.211	[M-H]-1	1.10E+07	Glycosides
180	(+/-)13-HODE	C18 H32 O3	296.23314	43.133	[M-H]-1	1.07E+07	Organic acids
181	Hyperoside	C21 H20 O12	464.09276	24.858	[M-H]-1	1.05E+07	Flavonoids
182	Geraniin	C41 H28 O27	952.07612	23.035	[M-H]-1	1.05E+07	Phenols
183	(+)-Syringaresinol	C22 H26 O8	418.16016	25.521	[M-H]-1	1.05E+07	Lignans
184	Curcumenol	C15 H22 O2	234.16011	30.438	[M+H]+1	1.02E+07	Terpenoids
185	Licochalcone B	C16 H14 O5	286.08134	28.128	[M-H]-1	1.01E+07	Flavonoids
186	Tectorigenin	C16 H12 O6	300.06117	28.618	[M-H]-1	9.93E+06	Flavonoids
187	Xanthosine	C10 H12 N4 O6	284.07399	20.529	[M-H]-1	9.74E+06	Flavonoids
188	Medrysone	C22 H32 O3	344.23232	33.138	[M+H]+1	9.63E+06	Terpenoids
189	Isomeranzin	C15 H16 O4	260.10286	27.556	[M+H]+1	9.53E+06	Lactones
190	Methyl 4-hydroxy-3-methoxycinnamate	C11 H12 O4	208.07195	26.738	[M+H]+1	9.49E+06	Phenylpropanoids
191	Neodiosmin	C28 H32 O15	608.16936	25.555	[M+H]+1	9.40E+06	Flavonoids
192	2,2-Dimethylsuccinic acid	C6 H10 O4	146.05694	22.446	[M-H]-1	9.38E+06	Organic acids
193	Isorhamnetin-3-O-rutinoside	C28 H32 O16	624.16429	25.704	[M+H]+1	9.07E+06	Flavonoids
194	Phytosphingosine	C18 H39 N O3	317.29048	37.814	[M+H]+1	8.86E+06	Miscellaneous
195	Daidzin	C21 H20 O9	416.10772	24.216	[M+H]+1	8.50E+06	Flavonoids
196	α-Eleostearic acid	C18 H30 O2	278.2224	38.65	[M+H]+1	8.19E+06	Organic acids
197	3-Phenyllactic acid	C9 H10 O3	166.06181	24.782	[M-H]-1	8.16E+06	Organic acids
198	8-Shogaol	C19 H28 O3	304.20143	45.115	[M+H]+1	8.13E+06	Phenols
199	Genistein	C15 H10 O5	270.05093	30.913	[M-H]-1	7.56E+06	Flavonoids
200	Catechol	C6 H6 O2	110.03603	15.807	[M-H]-1	7.49E+06	Phenols
201	Isoquercitrin	C21 H20 O12	464.092	24.385	[M+H]+1	7.42E+06	Flavonoids
202	Dehydrodiisoeugenol	C20 H22 O4	326.14925	26.889	[M+H]+1	7.31E+06	Flavonoids
203	3,3',5,5'-Tetramethyldiphenoquinone	C16 H16 O2	240.11307	43.873	[M+H]+1	7.08E+06	Quinones
204	Asperulosidic acid	C18 H24 O12	432.1241	22.622	[M-H]-1	7.01E+06	Organic acids
205	Shikimic acid	C7 H10 O5	174.05202	2.35	[M-H]-1	6.72E+06	Organic acids
206	Calycosin	C16 H12 O5	284.06636	29.033	[M+H]+1	6.60E+06	Flavonoids
207	cis-Aconitic acid	C6 H6 O6	174.01556	20.877	[M-H]-1	6.54E+06	Miscellaneous
208	Orientin	C21 H20 O11	448.0975	23.922	[M-H]-1	6.48E+06	Flavonoids
209	Isoalantolactone	C15 H20 O2	232.14455	33.683	[M+H]+1	6.28E+06	Terpenoids
210	Homoorientin	C21 H20 O11	448.09785	23.7	[M-H]-1	5.60E+06	Flavonoids
211	Butyl benzoate	C11 H14 O2	178.09807	26.073	[M+H]+1	5.38E+06	Miscellaneous
212	Pinocembrin	C15 H12 O4	256.07134	28.11	[M+H]+1	5.17E+06	Flavonoids
213	Glycitein	C16 H12 O5	284.06639	29.491	[M+H]+1	5.13E+06	Flavonoids
214	(±)-Abscisic acid	C15 H20 O4	264.13444	25.074	[M-H]-1	5.11E+06	Terpenoids
215	Coniferylaldehyde	C10 H10 O3	178.06159	27.371	[M+H]+1	4.87E+06	Miscellaneous
216	Asiatic acid	C30 H48 O5	470.33649	46.481	[M+H]+1	4.67E+06	Terpenoids
217	4-(3,4-Dihydroxyphenyl)-6,7-dihydroxy-2-naphthoic acid	C17 H12 O6	312.06151	29.8	[M-H]-1	4.05E+06	Miscellaneous
218	Xanthine	C5 H4 N4 O2	152.03276	5.053	[M-H]-1	3.54E+06	Terpenoids
219	8-Desoxygartanin	C23 H24 O5	380.15962	45.652	[M+H]+1	3.37E+06	Phenols
220	Abscisic acid	C15 H20 O4	264.13441	27.655	[M-H]-1	2.08E+06	Terpenoids
221	Hexadecanedioic acid	C16 H30 O4	286.21254	40.793	[M-H]-1	1.96E+06	Organic acids
222	Rheic acid	C15 H8 O6	284.03026	29.911	[M-H]-1	1.79E+06	Quinones
223	2,3-Dihydro-1-benzofuran-2-carboxylic acid	C9 H8 O3	164.04629	27.445	[M-H]-1	1.56E+06	Organic acids

Table S3 Entry-blood components of WLD by UPLC-MS/MS.

Number	Name	Formula	Annot. DeltaMass [ppm]	Calc. MW	m/z	RT [min]	Reference Ion
1	trans-3-Indoleacrylic acid	C11 H9 N O2	1.59	187.06363	188.0709	17.486	[M+H]+1
2	Tryptophan	C11 H12 N2 O2	1.66	204.09022	205.09749	17.488	[M+H]+1
3	Erucamide	C22 H43 N O	1.43	337.33495	338.34222	51.718	[M+H]+1
4	Indole	C8 H7 N	1.67	117.05805	118.06533	17.492	[M+H]+1
5	Cholic acid	C24 H40 O5	1.04	408.288	407.28068	32.054	[M-H]-1
6	Indole-3-carboxaldehyde	C9 H7 N O	2.01	145.05306	146.06033	17.489	[M+H]+1
7	2-Naphthylamine	C10 H9 N	2.22	143.07382	144.0811	17.49	[M+H]+1
8	3-Indoxyl sulphate	C8 H7 N O4 S	1.05	213.0098	212.00253	17.941	[M-H]-1
9	L-(-)-Methionine	C5 H11 N O2 S	2.21	149.05138	150.05866	2.653	[M+H]+1
10	Docosahexaenoic acid ethyl ester	C24 H36 O2	1.49	356.27206	357.27934	33.036	[M+H]+1
11	L-Tyrosine	C9 H11 N O3	2.66	181.07437	182.08167	5.497	[M+H]+1
12	Oleamide	C18 H35 N O	1.53	281.27229	282.27957	47.132	[M+H]+1
13	Arachidonic acid	C20 H32 O2	1.49	304.24068	305.24799	46.811	[M+H]+1
14	1,5-Naphthalenediamine	C10 H10 N2	1.66	158.08466	203.08286	17.483	[M+FA-H]-1
15	Corticosterone	C21 H30 O4	1.62	346.21497	347.22226	28.371	[M+H]+1
16	Docosahexaenoic acid	C22 H32 O2	1.37	328.24068	329.24798	46.544	[M+H]+1
17	α-Eleostearic acid	C18 H30 O2	1.9	278.22511	279.23239	45.868	[M+H]+1
18	Deoxycholic acid	C24 H40 O4	1.2	392.29313	391.28586	37.277	[M-H]-1
19	Bis(4-ethylbenzylidene)sorbitol	C24 H30 O6	1.65	414.20492	415.2122	35.393	[M+H]+1
20	Creatine	C4 H9 N3 O2	2.62	131.06982	132.07707	1.59	[M+H]+1
21	Arginine	C6 H14 N4 O2	2.84	174.11217	175.11945	1.788	[M+H]+1
22	Isoquinoline	C9 H7 N	2.09	129.05812	130.0654	25.628	[M+H]+1
23	Nalidixic Acid	C12 H12 N2 O3	1	232.08502	231.07768	21.921	[M-H]-1
24	Nicotinamide	C6 H6 N2 O	1.75	122.04823	123.0555	3.25	[M+H]+1
25	3-Indolepropionic acid	C11 H11 N O2	1.72	189.0793	190.08658	25.625	[M+H]+1
26	Glycocholic acid	C26 H43 N O6	1.19	465.30959	464.30222	29.102	[M-H]-1
27	4-Dodecylbenzenesulfonic acid	C18 H30 O3 S	0.77	326.19182	325.18454	44.122	[M-H]-1
28	(+/-)12(13)-DiHOME	C18 H34 O4	1.44	314.24616	313.23886	35.461	[M-H]-1
29	Betaine	C5 H11 N O2	2.01	117.07921	118.08645	1.561	[M+H]+1
30	Skatole	C9 H9 N	2.15	131.07378	132.08106	17.491	[M+H]+1
31	DL-Carnitine	C7 H15 N O3	2.3	161.10556	162.11283	1.57	[M+H]+1
32	Ursodeoxycholic acid	C24 H40 O4	1.25	392.29315	437.29133	33.036	[M+FA-H]-1

33	Pantothenic acid	C9 H17 N O5	1.3	219.11096	218.10354	16.518	[M-H]-1
34	Palmitoylcarnitine	C23 H45 N O4	1.38	399.33541	400.34269	40.156	[M+H]+1
35	3-Methylhippuric acid	C10 H11 N O3	1.5	193.07418	192.06689	20.992	[M-H]-1
36	Uric acid	C5 H4 N4 O3	1.31	168.02856	167.02122	3.698	[M-H]-1
37	Cortodoxone	C21 H30 O4	1.66	346.21499	347.22226	27.029	[M+H]+1
38	2-Phenylacetamide	C8 H9 N O	2.66	135.06877	136.07605	5.494	[M+H]+1
39	Creatinine	C4 H7 N3 O	2.53	113.0592	114.06647	1.863	[M+H]+1
40	Glycoursodeoxycholic acid	C26 H43 N O5	1.43	449.31477	448.3074	33.235	[M-H]-1
41	Hexadecanamide	C16 H33 N O	0.87	255.25644	256.26371	46.621	[M+H]+1
42	Pipecolic acid	C6 H11 N O2	1.97	129.07923	130.08651	2.429	[M+H]+1
43	DL-Stachydrine	C7 H13 N O2	1.85	143.09489	144.10217	2.041	[M+H]+1
44	Choline	C5 H13 N O	1.89	103.09991	104.10719	1.533	[M+H]+1
45	Taurochenodeoxycholic acid	C26 H45 N O6 S	1.19	499.29735	498.29008	30.278	[M-H]-1
46	Bilirubin	C33 H36 N4 O6	0.37	584.2637	585.27092	50.636	[M+H]+1
47	Phenylacetylglycine	C10 H11 N O3	1.93	193.07427	194.08161	19.976	[M+H]+1
48	Hippuric acid	C9 H9 N O3	1.62	179.05853	180.0659	18.916	[M+H]+1
49	Hecogenin	C27 H42 O4	1.53	430.30897	431.31642	40.342	[M+H]+1
50	6-Methoxyquinoline	C10 H9 N O	2.21	159.06877	160.07604	17.495	[M+H]+1
51	Dodecyl sulfate	C12 H26 O4 S	0.58	266.15534	265.14806	38.748	[M-H]-1
52	Riboflavin	C17 H20 N4 O6	1.71	376.13893	377.14621	19.803	[M+H]+1
53	D-(+)-Galactose	C6 H12 O6	1.37	180.06363	179.05636	1.499	[M-H]-1
54	1-Linoleoyl glycerol	C21 H38 O4	1.71	354.27761	355.28489	45.886	[M+H]+1
55	Cytidine	C9 H13 N3 O5	2.07	243.08602	244.0933	2.719	[M+H]+1
56	N3,N4-Dimethyl-L-arginine	C8 H18 N4 O2	1.67	202.14331	203.15059	1.982	[M+H]+1
57	5-Methylcytosine	C5 H7 N3 O	2.29	125.0592	126.06647	5.432	[M+H]+1
58	Arachidonic acid methyl ester	C21 H34 O2	1.01	318.2562	319.26348	49.268	[M+H]+1
59	Indole-3-acrylic acid	C11 H9 N O2	1.15	187.06354	186.05623	25.045	[M-H]-1
60	Citric acid	C6 H8 O7	0.94	192.02718	191.01991	1.689	[M-H]-1
61	4-Hydroxybutyric acid (GHB)	C4 H8 O3	0.68	104.04741	103.04014	2.676	[M-H]-1
62	Nor-9-carboxy-δ9-THC	C21 H28 O4	1.58	344.1993	345.20658	27.648	[M+H]+1
63	Oleoyl ethanolamide	C20 H39 N O2	2	325.29873	326.306	46.064	[M+H]+1
64	Pseudouridine	C9 H12 N2 O6	1.09	244.0698	243.06245	2.793	[M-H]-1
65	Bufalin	C24 H34 O4	1.06	386.24612	387.2534	31.553	[M+H]+1
66	5-Methylcytidine	C10 H15 N3 O5	3.28	257.10201	258.10907	5.437	[M+H]+1
67	Acetyl-β-methylcholine	C8 H17 N O2	2.04	159.12625	160.13353	2.647	[M+H]+1
68	DL-4-Hydroxyphenyllactic acid	C9 H10 O4	1.41	182.05816	181.05089	17.586	[M-H]-1
69	4-Hydroxyquinoline	C9 H7 N O	1.44	145.05297	146.06033	20.744	[M+H]+1

70	Docosanamide	C22 H45 N O	1.19	339.35052	340.35779	55.568	[M+H]+1
71	D-Sphingosine	C18 H37 N O2	1.78	299.28296	300.29024	36.002	[M+H]+1
72	Bis(2-ethylhexyl) phthalate	C24 H38 O4	1.01	390.2774	391.28468	50.186	[M+H]+1
73	Carbaryl	C12 H11 N O2	2.09	201.0794	202.08668	17.635	[M+H]+1
74	Proline	C5 H9 N O2	2.47	115.06361	116.07084	1.59	[M+H]+1
75	gamma-Glutamylleucine	C11 H20 N2 O5	1.81	260.13769	261.14506	17.723	[M+H]+1
76	Aspartame	C14 H18 N2 O5	1.94	294.12214	295.12943	18.5	[M+H]+1
77	4-Phenylbutyric acid	C10 H12 O2	2.3	164.08411	165.09138	29.68	[M+H]+1
78	Palmitoleic acid	C16 H30 O2	1.34	254.22492	255.23222	46.57	[M+H]+1
79	Spermidine	C7 H19 N3	2.43	145.15825	146.16553	1.828	[M+H]+1
80	Progesterone	C21 H30 O2	1.96	314.2252	315.23247	38.274	[M+H]+1
81	Dihydrosphingosine	C18 H39 N O2	1.83	301.29863	302.30591	36.923	[M+H]+1
82	4-Hydroxybenzaldehyde	C7 H6 O2	2.22	122.03705	123.04433	5.497	[M+H]+1
83	Stearamide	C18 H37 N O	1.1	283.28783	284.2951	48.969	[M+H]+1
84	Docosahexaenoic acid methyl ester	C23 H34 O2	1.23	342.2563	343.26358	48.763	[M+H]+1
85	3-Phenyllactic acid	C9 H10 O3	0.96	166.06315	165.05588	21.098	[M-H]-1
86	Hexanoylcarnitine	C13 H25 N O4	2.13	259.17891	260.18619	21.379	[M+H]+1
87	Octadecanedioic acid	C18 H34 O4	0.88	314.24599	313.23871	42.028	[M-H]-1
88	3-Methylhistidine	C7 H11 N3 O2	2.37	169.08553	170.0928	1.805	[M+H]+1
89	Coumarin	C9 H6 O2	2.27	146.03711	147.04439	5.491	[M+H]+1
90	1-Methylnicotinamide	C7 H8 N2 O	2.68	136.06403	137.0713	1.85	[M+H]+1
91	2-Mercaptobenzothiazole	C7 H5 N S2	1.99	166.98667	167.99395	27.199	[M+H]+1
92	Uridine	C9 H12 N2 O6	0.8	244.06973	243.06245	5.341	[M-H]-1
93	3,5-di-tert-Butyl-4-hydroxybenzoic acid	C15 H22 O3	1.27	250.15721	251.16457	35.874	[M+H]+1
94	Cholest-4-en-3-one	C27 H44 O	0.73	384.33949	385.34677	59.753	[M+H]+1
95	Tridemorph	C19 H39 N O	1.18	297.30352	298.31079	48.679	[M+H]+1
96	Cytosine	C4 H5 N3 O	1.7	111.04345	112.05073	1.634	[M+H]+1
97	5'-S-Methyl-5'-thioadenosine	C11 H15 N5 O3 S	1.98	297.09015	298.09743	17.706	[M+H]+1
98	4'-(Imidazol-1-yl)acetophenone	C11 H10 N2 O	2.32	186.07974	187.08702	21.928	[M+H]+1
99	β-Asarone	C12 H16 O3	1.88	208.11034	209.11761	35.703	[M+H]+1
100	Palmitoyl ethanolamide	C18 H37 N O2	1.81	299.28297	300.29025	45.478	[M+H]+1
101	Stearoyl ethanolamide	C20 H41 N O2	1.25	327.31414	328.32142	48.063	[M+H]+1
102	8-Hydroxyquinoline	C9 H7 N O	1.82	145.05303	146.0603	21.375	[M+H]+1
103	Thymine	C5 H6 N2 O2	2.74	126.04327	127.05055	16.204	[M+H]+1
104	Myristyl sulfate	C14 H30 O4 S	0.95	294.18676	293.17948	44.463	[M-H]-1

105	D-(-)-Glutamine	C5 H10 N2 O3	1.25	146.06932	145.06201	1.544	[M-H]-1
106	(R)-Equol	C15 H14 O3	1.85	242.09474	243.10202	22.918	[M+H]+1
107	Anserine	C10 H16 N4 O3	1.98	240.12272	241.12999	1.824	[M+H]+1
108	3-Hydroxydecanoic acid	C10 H20 O3	1.23	188.14148	187.1342	30.091	[M-H]-1
109	Isoalantolactone	C15 H20 O2	1.64	232.14671	233.15399	30.625	[M+H]+1
110	Thymidine	C10 H14 N2 O5	1.47	242.09063	241.08332	16.202	[M-H]-1
111	Levothyroxine	C15 H11 I4 N O4	1.54	776.68788	777.69517	28.651	[M+H]+1
112	trans-Anethole	C10 H12 O	2.51	148.08919	149.09646	37.449	[M+H]+1
113	L-Ascorbic acid 2-sulfate	C6 H8 O9 S	0.99	255.98916	254.98188	2.294	[M-H]-1
114	18 β-Glycyrrhetintic Acid	C30 H46 O4	1.44	470.34029	471.34772	43.021	[M+H]+1
115	2-Oxindole	C8 H7 N O	2.08	133.05304	134.06032	21.011	[M+H]+1
116	Proline-hydroxyproline	C10 H16 N2 O4	1.78	228.11141	229.11869	2.381	[M+H]+1
117	2-Hydroxycaproic acid	C6 H12 O3	0.87	132.07876	131.07148	19.398	[M-H]-1
118	Homoarginine	C7 H16 N4 O2	2.33	188.12776	189.13504	1.843	[M+H]+1
119	Haloxyfop	C15 H11 Cl F3 N O4	1.19	361.0333	360.02598	36.717	[M-H]-1
120	Kynurenic acid	C10 H7 N O3	2.05	189.04298	190.05029	18.45	[M+H]+1
121	Docosapentaenoic acid	C22 H34 O2	1.32	330.25631	331.26359	47.147	[M+H]+1
122	9-Oxo-ODE	C18 H30 O3	1.61	294.21997	295.22724	39.6	[M+H]+1
123	3-tert-Butyladipic acid	C10 H18 O4	1.47	202.12081	201.11355	20.487	[M-H]-1
124	CMPF	C12 H16 O5	1.1	240.10004	239.09275	25.178	[M-H]-1
125	Eicosapentaenoic acid methyl ester	C21 H32 O2	1.87	316.24082	317.2481	45.816	[M+H]+1
126	Ethyl myristate	C16 H32 O2	-3.23	256.2394	255.23213	41.135	[M-H]-1
127	Oxadixyl	C14 H18 N2 O4	2.06	278.12723	279.13456	17.019	[M+H]+1
128	2-Arachidonoyl glycerol	C23 H38 O4	1.82	378.2777	379.28497	45.696	[M+H]+1
129	Cortisone	C21 H28 O5	0.94	360.19401	361.20129	27.103	[M+H]+1
130	11,12-Epoxy-(5Z,8Z,11Z)-icosatrienoic acid	C20 H32 O3	0.53	320.23531	319.22804	41.7	[M-H]-1
131	Linolenic acid ethyl ester	C20 H34 O2	1.42	306.25631	307.26359	47.629	[M+H]+1
132	Osthol	C15 H16 O3	1.12	244.11022	245.11756	28.795	[M+H]+1
133	4-Quinolinecarboxylic acid	C10 H7 N O2	1.7	173.04797	174.05527	20.731	[M+H]+1
134	3-(4-Hydroxyphenyl)propionic acid	C9 H10 O3	0.51	166.06308	165.0558	17.363	[M-H]-1
135	Isoliquiritigenin	C15 H12 O4	2.05	256.07408	257.08136	21.833	[M+H]+1

136	2,6-Di-tert-butyl-1,4-benzoquinone	C14 H20 O2	1.6	220.14668	221.15396	33.038	[M+H]+1
137	Adenosine 5'-monophosphate	C10 H14 N5 O7 P	1.31	347.06354	348.07097	3.207	[M+H]+1
138	Hydroxyproline	C5 H9 N O3	2.39	131.05856	132.06583	1.987	[M+H]+1
139	3-Methyloxyindole	C9 H9 N O	1.69	147.06866	148.076	20.998	[M+H]+1
140	Biotin	C10 H16 N2 O3 S	1.97	244.08864	245.09592	19.777	[M+H]+1
141	Indole-3-acetic acid	C10 H9 N O2	2.34	175.06374	176.07101	23.5	[M+H]+1
142	Clareolide	C16 H26 O2	1.36	250.19362	251.2009	44.046	[M+H]+1
143	1-Methyladenosine	C11 H15 N5 O4	1.66	281.11287	282.12015	5.606	[M+H]+1
144	17-Octadecynoic acid	C18 H32 O2	0.92	280.24049	279.23321	37.542	[M-H]-1
145	1H-Imidazole-4-carboxylic acid	C4 H4 N2 O2	1.75	112.02747	113.03475	2.707	[M+H]+1
146	4-(4-Nitrobenzyl)pyridine	C12 H10 N2 O2	2.07	214.07467	215.08195	21.921	[M+H]+1
147	L-Phenylalanine	C9 H11 N O2	2.52	165.07939	166.08667	20.998	[M+H]+1
148	Salicylic acid	C7 H6 O3	0.86	138.03181	137.02454	23.335	[M-H]-1
149	Indole-3-lactic acid	C11 H11 N O3	2.05	205.07431	206.08159	22.047	[M+H]+1
150	N-Acetyl-DL-tryptophan	C13 H14 N2 O3	1.01	246.10069	245.09338	22.2	[M-H]-1
151	Histidine	C6 H9 N3 O2	2.57	155.06987	156.07715	1.726	[M+H]+1
152	Tetradecanedioic acid	C14 H26 O4	1.32	258.18345	257.17617	33.653	[M-H]-1
153	Dodecanedioic acid	C12 H22 O4	0.56	230.15194	229.14466	28.751	[M-H]-1
154	N6,N6,N6-Trimethyl-L-lysine	C9 H20 N2 O2	-3.92	188.15174	227.11433	1.817	[M+K]+1
155	Xanthurenic acid	C10 H7 N O4	2.72	205.03807	206.04534	17.975	[M+H]+1
156	N-Acetyl-D-alloisoleucine	C8 H15 N O3	1.63	173.10548	172.09816	20.457	[M-H]-1
157	Quillaic acid	C30 H46 O5	1.18	486.3351	485.32782	41.426	[M-H]-1
158	16-Hydroxyhexadecanoic acid	C16 H32 O3	0.7	272.23533	271.22806	39.071	[M-H]-1
159	3,4'-Dihydroxy-5,5'-dimethoxybibenzyl	C16 H18 O4	0.5	274.12065	275.12792	28.758	[M+H]+1
160	Ginkgolic acid C17-1	C24 H38 O3	1.51	374.28266	375.28989	36.678	[M+H]+1
161	N-Acetyl-L-phenylalanine	C11 H13 N O3	1.52	207.08986	206.08251	21.465	[M-H]-1
162	Artemisinic acid	C15 H22 O2	1.64	234.16236	235.16964	46.804	[M+H]+1
163	17α-Hydroxyprogesterone	C21 H30 O3	1.53	330.22	331.22728	32.313	[M+H]+1
164	L-Leucine	C6 H13 N O2	2.09	131.0949	132.10218	19.865	[M+H]+1
165	Ethyl palmitoleate	C18 H34 O2	1.21	282.25622	283.26355	48.351	[M+H]+1
166	17α-Methyl-androstan-3-hydroxyimine-17β-ol	C20 H33 N O2	1.67	319.25166	320.25894	45.893	[M+H]+1
167	(R)-3-Hydroxy myristic acid	C14 H28 O3	0.19	244.20389	243.19661	40.422	[M-H]-1

168	6-Methylquinoline	C10 H9 N	2.22	143.07382	144.08109	18.894	[M+H]+1
169	Pyrethrini	C21 H28 O3	1.58	328.20436	329.21164	28.37	[M+H]+1
170	Indole-3-carboxylic acid	C9 H7 N O2	2.25	161.04804	162.05532	18.453	[M+H]+1
171	4-Ethylphenol	C8 H10 O	0.04	122.07317	121.06589	23.19	[M-H]-1
172	Indole-3-carbinol	C9 H9 N O	2.08	147.06872	148.076	22.717	[M+H]+1
173	9-Oxo-10(E),12(E)-octadecadienoic acid	C18 H30 O3	1.7	294.22	295.22727	40.913	[M+H]+1
174	Nandrolone	C18 H26 O2	1.58	274.19371	275.20099	33.038	[M+H]+1
175	(+/-)-Gingerol	C17 H26 O4	1.13	294.18344	293.17617	35.307	[M-H]-1
176	β-Asarone	C12 H16 O3	1.61	208.11028	209.11756	25.181	[M+H]+1
177	Dibutyl phthalate	C16 H22 O4	0.42	278.15193	277.14465	28.137	[M-H]-1
178	Dihydroroseoside	C19 H32 O8	1.54	388.21032	387.20304	24.519	[M-H]-1
179	Testosterone cypionate	C27 H40 O3	2.22	412.29866	413.30594	46.024	[M+H]+1
180	Terpestacin	C25 H38 O4	1.57	402.27764	403.28492	45.401	[M+H]+1
181	5-Hydroxyindole-3-acetic acid	C10 H9 N O3	1.37	191.0585	192.06578	20.207	[M+H]+1
182	4-Methoxybenzaldehyde	C8 H8 O2	1.92	136.05269	137.05997	26.485	[M+H]+1
183	L-2-Aminoadipic acid	C6 H11 N O4	1.96	161.06912	162.0764	63.465	[M+H]+1
184	Epitestosterone	C19 H28 O2	1.06	288.20924	289.21651	31.595	[M+H]+1
185	Gitogenin	C27 H44 O4	3.39	432.32543	433.33214	34.572	[M+H]+1
186	Tauroursodeoxycholic acid	C26 H45 N O6 S	2.1	499.29781	500.30508	30.924	[M+H]+1
187	N-Tigloylglycine	C7 H11 N O3	0.31	157.07394	156.06667	17.146	[M-H]-1
188	5-(2-Nitroprop-1-enyl)-1,3-benzodioxole	C10 H9 N O4	2.16	207.05361	208.06088	18.779	[M+H]+1
189	Gentian violet	C25 H29 N3	2.01	371.23689	372.24417	34.665	[M+H]+1
190	Methyl linoleate	C19 H34 O2	1.07	294.2562	295.26347	49.659	[M+H]+1
191	2,4-Dihydroxybenzoic acid	C7 H6 O4	1.05	154.02677	153.01949	19.372	[M-H]-1
192	Stercobilin	C33 H46 N4 O6	2.45	594.34319	595.3504	24.965	[M+H]+1
193	L-Histidine	C6 H9 N3 O2	1.25	155.06967	154.06239	1.489	[M-H]-1
194	Trenbolone	C18 H22 O2	1.45	270.16237	271.16965	29.885	[M+H]+1
195	5-Henicosyl-1,3-benzenediol	C27 H48 O2	1.17	404.36591	405.37318	50.877	[M+H]+1
196	2-Furoic acid	C5 H4 O3	0.37	112.01609	111.00881	3.348	[M-H]-1
197	N-Arachidonoyl taurine	C22 H37 N O4 S	1.03	411.24475	410.23748	42.833	[M-H]-1
198	Diosgenin	C27 H42 O3	0.36	414.31354	415.32082	39.081	[M+H]+1
199	2,4-Dimethylbenzaldehyde	C9 H10 O	1.16	134.07332	135.0806	35.444	[M+H]+1
200	Dibutyl Succinate	C12 H22 O4	0.8	230.15199	229.14472	20.647	[M-H]-1
201	N-Butylbenzenesulfonamide	C10 H15 N O2 S	1.94	213.08276	214.09004	30.884	[M+H]+1
202	Folinic acid	C20 H23 N7 O7	2.74	473.16719	474.17447	16.933	[M+H]+1

203	Azelaic acid	C9 H16 O4	0.93	188.10503	187.09776	23.114	[M-H]-1
204	10-HDA	C10 H18 O3	0.58	186.1257	185.11843	29.718	[M-H]-1
205	Monobutyl phthalate	C12 H14 O4	0.56	222.08933	221.08206	29.6	[M-H]-1
206	Medicagenic acid	C30 H46 O6	1.84	502.33036	501.323	33.321	[M-H]-1
207	Jasmonic acid	C12 H18 O3	2.05	210.12602	211.1333	34.766	[M+H]+1
208	Norharman	C11 H8 N2	2.05	168.06909	169.07637	21.932	[M+H]+1
209	DL-Tryptophan	C11 H12 N2 O2	1.67	204.09022	205.0975	21.919	[M+H]+1
210	Docosapentaenoic acid methyl ester	C23 H36 O2	0.78	344.2718	345.27908	49.447	[M+H]+1
211	p-Hydroxybenzaldehyde	C7 H6 O2	1.63	122.03698	123.04425	22.919	[M+H]+1
212	12-Aminododecanoic acid	C12 H25 N O2	2.2	215.189	216.19629	26.563	[M+H]+1
213	Nabumetone	C15 H16 O2	1.95	228.11547	229.12275	29.898	[M+H]+1
214	Sarsasapogenin	C27 H44 O3	1.81	416.3298	417.33707	40.886	[M+H]+1
215	3,4-Dimethylbenzoic acid	C9 H10 O2	1.52	150.06831	151.07558	28.01	[M+H]+1
216	Kaempferol-7-O-glucoside	C21 H20 O11	1.48	448.10122	447.09387	22.94	[M-H]-1
217	D-α-Tocopherol	C29 H50 O2	1.79	430.38185	431.38913	47.352	[M+H]+1
218	Boldine	C19 H21 N O4	1.19	327.14745	328.15472	19.887	[M+H]+1
219	3-O-β-D-Glucopyranosylandrographolide	C26 H40 O10	1.23	512.26278	511.2555	32.913	[M-H]-1
220	α-Asarone	C12 H16 O3	1.62	208.11028	209.11756	28.49	[M+H]+1
221	Cyclo(Leu-Pro)	C11 H18 N2 O2	2.12	210.13727	211.14455	21.057	[M+H]+1
222	Hexanoylglycine	C8 H15 N O3	1.71	173.10549	172.09816	21.297	[M-H]-1
223	Succinic acid	C4 H6 O4	0.81	118.0267	117.01943	4.143	[M-H]-1
224	9S,13R-12-Oxophytodienoic acid	C18 H28 O3	1.46	292.20427	293.21155	38.229	[M+H]+1
225	Tetradecanedioic acid	C14 H26 O4	1.32	258.18345	257.17617	32.789	[M-H]-1
226	Aprobarbital	C10 H14 N2 O3	2.26	210.10092	211.10819	16.062	[M+H]+1
227	all-cis-4,7,10,13,16-Docosapentaenoic acid	C22 H34 O2	1.23	330.25628	329.24898	47.542	[M-H]-1
228	Mesaconic acid	C5 H6 O4	1.3	130.02678	129.0195	3.989	[M-H]-1
229	Testosterone 17-Isocaproate	C25 H38 O3	1.65	386.28273	387.29001	34.612	[M+H]+1
230	Testosterone decanoate	C29 H46 O3	1.56	442.34538	443.35266	40.629	[M+H]+1
231	Monoolein	C21 H40 O4	1.42	356.29316	357.30044	47.417	[M+H]+1
232	Adrenic acid	C22 H36 O2	1.12	332.2719	333.27918	48.071	[M+H]+1
233	3-Hydroxysebacic acid	C10 H18 O5	0.73	218.11558	217.10831	23.531	[M-H]-1
234	Senkyunolide H	C12 H16 O4	1.64	224.10523	225.1125	29.457	[M+H]+1
235	Mono(2-ethylhexyl) phthalate (MEHP)	C16 H22 O4	0.57	278.15197	277.14469	38.778	[M-H]-1

236	Biliverdin	C33 H34 N4 O6	2.13	582.24908	581.24162	31.716	[M-H]-1
237	Cortisol	C21 H30 O5	1.47	362.20986	363.21722	24.277	[M+H]+1
238	Epinephrine	C9 H13 N O3	2.61	183.09002	184.0973	16.52	[M+H]+1
239	N-Hydroxy MDA	C10 H13 N O3	2.45	195.09002	196.0973	17.083	[M+H]+1
240	Testosterone propionate	C22 H32 O3	1.27	344.23558	345.24286	27.186	[M+H]+1
241	(±)-Abscisic acid	C15 H20 O4	1.45	264.13654	265.14382	28.003	[M+H]+1
242	4-Indolecarbaldehyde	C9 H7 N O	1.1	145.05292	144.04556	22.883	[M-H]-1
243	(-)-Camphanic acid	C10 H14 O4	1.21	198.08945	197.08217	22.32	[M-H]-1
244	Pyrethrin I	C21 H28 O3	1.77	328.20443	329.2117	27.031	[M+H]+1
245	Phthaldialdehyde	C8 H6 O2	2.31	134.03709	135.04437	21.558	[M+H]+1
246	N4-Acetylcytidine	C11 H15 N3 O6	1.47	285.0965	284.08905	16.516	[M-H]-1
247	(+)-Magnoflorine	C20 H23 N O4	1.16	341.1631	342.17038	20.113	[M+H]+1
248	6-Gingerol	C17 H26 O4	1.18	294.18346	293.17618	32.879	[M-H]-1
249	Suberic acid	C8 H14 O4	0.78	174.08934	173.08207	21.233	[M-H]-1
250	N-Cinnamoylglycine	C11 H11 N O3	1.21	205.07414	204.0668	22.814	[M-H]-1
251	Diisopropyl phthalate	C14 H18 O4	1.26	250.12082	251.1281	28.497	[M+H]+1
252	Dehydrocostus lactone	C15 H18 O2	1.64	230.13106	231.13833	29.902	[M+H]+1
253	Sedanolide	C12 H18 O2	1.96	194.13106	195.13834	28.487	[M+H]+1
254	Linderalactone	C15 H16 O3	1.8	244.11038	245.11766	30.572	[M+H]+1
255	3-Methyladipic acid	C7 H12 O4	1.06	160.07373	159.06645	18.333	[M-H]-1
256	2,3-Dinor-8-epi-prostaglandin F2α	C18 H30 O5	0.87	326.20961	325.20233	37.557	[M-H]-1
257	Primobolan	C27 H42 O3	1.34	414.31395	415.32123	36.629	[M+H]+1
258	3,5-Dimethyl-4- methoxybenzoic acid	C10 H12 O3	1.39	180.07889	181.08617	28.627	[M+H]+1
259	Perillartine	C10 H15 N O	2.64	165.1158	166.12308	16.528	[M+H]+1
260	N2,N2-Dimethylguanosine	C12 H17 N5 O5	1.96	311.12358	312.13092	16.987	[M+H]+1
261	2-Methyl-6-phenylpyrimidin-4-ol	C11 H10 N2 O	2.15	186.07971	187.08699	18.892	[M+H]+1
262	(+/-)-Cannabichromeorcin	C17 H22 O2	1.63	258.1624	259.16968	26.483	[M+H]+1
263	Prostaglandin E2	C20 H32 O5	1.04	352.22534	351.21806	31.091	[M-H]-1
264	Flavin mononucleotide (FMN)	C17 H21 N4 O9 P	1.77	456.10542	455.09802	19.052	[M-H]-1
265	3,4-EDMA	C12 H17 N O2	2.22	207.12639	208.13366	16.493	[M+H]+1
266	2-Naphthalenesulfonic acid	C10 H8 O3 S	0.88	208.0196	207.01232	21.147	[M-H]-1
267	2-Hydroxymyristic acid	C14 H28 O3	0.79	244.20404	243.19676	34.636	[M-H]-1
268	Honokiol	C18 H18 O2	1.41	266.13106	265.12378	38.653	[M-H]-1
269	Cyclo(phenylalanyl-prolyl)	C14 H16 N2 O2	1.79	244.12161	245.12889	21.908	[M+H]+1

270	1-Naphthol	C10 H8 O	1.89	144.05779	145.06506	26.487	[M+H]+1
271	Luvangetin	C15 H14 O4	1.92	258.08971	259.09698	29.891	[M+H]+1
272	L-Isoleucine	C6 H13 N O2	1.17	131.09478	130.08748	20.47	[M-H]-1
273	N-Phenylacetylglutamine	C13 H16 N2 O4	1.32	264.11135	263.10394	19.257	[M-H]-1
274	Rimantadine	C12 H21 N	1.55	179.16768	180.17495	28.15	[M+H]+1
275	Apigenin 7-O-glucuronide	C21 H18 O11	1.81	446.08572	445.07835	22.257	[M-H]-1
276	5-Methyldeoxycytidine	C10 H15 N3 O4	1.51	241.10662	240.0993	6.667	[M-H]-1
277	4-Acetamidobenzaldehyde	C9 H9 N O2	1.92	163.06364	164.07092	17.817	[M+H]+1
278	Harmine	C13 H12 N2 O	2.2	212.09543	213.10271	19.257	[M+H]+1
279	cis-12-Octadecenoic acid methyl ester	C19 H36 O2	0.81	296.27177	297.27905	51.305	[M+H]+1
280	β-D-Glucopyranuronic acid	C6 H10 O7	0.97	194.04284	193.03556	20.47	[M-H]-1
281	(+/-)14(15)-DiHET	C20 H34 O4	0.63	338.24592	337.23865	36.622	[M-H]-1
282	Methylphenidate	C14 H19 N O2	1.92	233.14203	234.1493	22.192	[M+H]+1
283	4-Ethoxybenzaldehyde	C9 H10 O2	2.08	150.06839	151.07567	24.202	[M+H]+1
284	Medrysone	C22 H32 O3	1.27	344.23558	345.24286	26.373	[M+H]+1
285	N-Acetyl-L-tyrosine	C11 H13 N O4	1.18	223.08472	222.07736	18.086	[M-H]-1
286	4-Nitrophenol	C6 H5 N O3	0.88	139.02707	138.01979	23.522	[M-H]-1
287	(2Z)-2-Octyl-2-pentenedioic acid	C13 H22 O4	0.94	242.15204	241.14476	29.514	[M-H]-1
288	4-Coumaric acid	C9 H8 O3	1.26	164.04755	163.04027	21.289	[M-H]-1
289	Cafestol	C20 H28 O3	1.42	316.20429	317.21157	29.782	[M+H]+1
290	Myristic acid alkyne	C14 H24 O2	1.14	224.17789	223.17061	33.624	[M-H]-1
291	Lupenone	C30 H48 O	1.83	424.37129	425.37857	48.117	[M+H]+1
292	Phthalic Acid, Bis-Hexyl Ester	C20 H30 O4	1.25	334.21483	333.20755	35.049	[M-H]-1
293	Parthenolide	C15 H20 O3	1.05	248.14151	249.14878	26.149	[M+H]+1
294	Xanthosine	C10 H12 N4 O6	1.78	284.07619	283.06882	15.979	[M-H]-1
295	Dinoseb	C10 H12 N2 O5	1.3	240.07493	239.06766	34.879	[M-H]-1
296	Isobornyl methacrylate	C14 H22 O2	1.73	222.16236	223.16964	28.136	[M+H]+1
297	Curcumol	C15 H24 O2	1.29	236.17793	237.18521	26.614	[M+H]+1
298	Acetaminophen glucuronide	C14 H17 N O8	1.42	327.09588	326.08861	18.416	[M-H]-1
299	Wogonoside	C22 H20 O11	1.78	460.10138	461.10872	20.913	[M+H]+1
300	4-Hydroxycoumarin	C9 H6 O3	1.11	162.03187	161.0246	23.582	[M-H]-1
301	15-Deoxy-δ12,14 -Prostaglandin J2	C20 H28 O3	0.28	316.20393	315.19666	27.112	[M-H]-1
302	3-Methoxybenzaldehyde	C8 H8 O2	1.92	136.05269	137.05997	25.429	[M+H]+1
303	Lusitanicoside	C21 H30 O10	1.28	442.18446	441.17719	27.553	[M-H]-1
304	Methyl cinnamate	C10 H10 O2	1.93	162.06839	163.07567	26.131	[M+H]+1

305	11-Hydroxy-δ(9)-THC	C21 H30 O3	1.77	330.22008	331.22736	26.207	[M+H]+1
306	Mussaenosidic acid	C16 H24 O10	4.82	376.13876	375.13143	19.333	[M-H]-1
307	Bavachinin	C21 H22 O4	-2.43	338.15099	339.15826	42.748	[M+H]+1
308	Glycitein	C16 H12 O5	1.84	284.069	285.07627	25.219	[M+H]+1
309	Betaxolol	C18 H29 N O3	2.58	307.21554	615.43842	23.081	[2M+H]+1
310	Glycodeoxycholic acid	C26 H43 N O5	1.17	449.31465	448.30737	31.286	[M-H]-1
311	5α-Dihydrotestosterone glucuronide	C25 H38 O8	1.44	466.25734	465.25006	26.311	[M-H]-1
312	CAPSO	C9 H19 N O4 S	0.53	237.10361	236.09633	21.516	[M-H]-1
313	2-Hydroxycinnamic acid	C9 H8 O3	0.66	164.04745	163.04018	17.577	[M-H]-1
314	5-Sulfosalicylic acid	C7 H6 O6 S	0.77	217.98868	216.9814	16.129	[M-H]-1
315	Altrenogest	C21 H26 O2	1.2	310.19365	311.20093	28.372	[M+H]+1
316	Atractylodin	C13 H10 O	2.53	182.07363	183.0809	34.667	[M+H]+1
317	Senkyunolide A	C12 H16 O2	2.35	192.11548	193.12276	19.876	[M+H]+1
318	Oxazepam	C15 H11 Cl N2 O2	-0.6	286.05073	285.04346	26.394	[M-H]-1
319	Pinolenic acid	C18 H30 O2	2.25	278.22521	279.23248	28.126	[M+H]+1
320	Palmitelaidic acid methyl ester	C17 H32 O2	0.53	268.24037	269.24765	49.413	[M+H]+1
321	Trolox	C14 H18 O4	1.31	250.12084	251.12811	28.798	[M+H]+1
322	Pimelic acid	C7 H12 O4	0.68	160.07367	159.06639	19.168	[M-H]-1
323	4-Guanidinobutyric acid	C5 H11 N3 O2	2.13	145.08544	146.09271	2.69	[M+H]+1
324	Methiocarb-sulfone	C11 H15 N O4 S	1.07	257.07245	256.06516	19.994	[M-H]-1
325	2'-O-Methyluridine	C10 H14 N2 O6	2.15	258.08574	259.09302	2.399	[M+H]+1
326	Xylenesulfonate	C8 H10 O3 S	0.56	186.03517	185.02789	19.61	[M-H]-1
327	Heptanophenone	C13 H18 O	1.65	190.13608	191.14336	48.057	[M+H]+1
328	Laurolactam	C12 H23 N O	1.63	197.17829	198.18556	48.057	[M+H]+1
329	Diethanolamine	C4 H11 N O2	2.86	105.07928	106.08656	63.446	[M+H]+1
330	Phenylacetaldehyde	C8 H8 O	1.02	120.05764	165.05585	21.155	[M+FA-H]-1
331	N-Formylmethionine	C6 H11 N O3 S	1.09	177.04616	176.03888	16.673	[M-H]-1
332	6-Methylnicotinamide	C7 H8 N2 O	2.35	136.06398	137.07126	1.577	[M+H]+1
333	trans-Aconitic acid	C6 H6 O6	0.05	174.01645	173.00917	1.691	[M-H]-1
334	4-Acetamidobenzoic acid	C9 H9 N O3	2.31	179.05866	180.06593	19.648	[M+H]+1
335	DL-Arginine	C6 H14 N4 O2	2.57	174.11212	175.1194	1.515	[M+H]+1
336	Methionine	C5 H11 N O2 S	2.29	149.05139	150.05867	1.719	[M+H]+1
337	Hexadecanedioic acid	C16 H30 O4	0.9	286.21467	285.20731	37.833	[M-H]-1
338	2'-Deoxycytidine	C9 H13 N3 O4	1.16	227.09087	272.08908	3.551	[M+FA-H]-1

339	N-Acetylglycine	C4 H7 N O3	0.2	117.04262	116.03534	3.295	[M-H]-1
340	4-tert-Butylcyclohexyl acetate	C12 H22 O2	2.05	198.16239	199.16966	45.267	[M+H]+1
341	Daidzein	C15 H10 O4	1.46	254.05828	255.06561	24.82	[M+H]+1
342	Decanoylcarnitine	C17 H33 N O4	1.92	315.24156	316.24884	29.337	[M+H]+1
343	Palmitic acid	C16 H32 O2	-2.61	256.23956	255.23228	43.94	[M-H]-1
344	Acetylarginine	C8 H16 N4 O3	2.4	216.12276	217.13004	2.827	[M+H]+1
345	Isoferulic acid	C10 H10 O4	0.45	194.058	193.05072	19.461	[M-H]-1
346	Oleic acid	C18 H34 O2	-3.38	282.25493	281.24765	43.244	[M-H]-1
347	Acetyl-L-carnitine	C9 H17 N O4	1.94	203.11615	204.12343	2.928	[M+H]+1
348	Noroxymorphone	C16 H17 N O4	1.85	287.11629	288.12357	32.401	[M+H]+1

Table S4 The specific herb information of WLD

Chinese name	Scientific name	Botanical name	Medicine parts	Place of Origin	Batch number	Amount (g)
Cangzhu	Atractylodis Rhizoma	*Atractylodes lancea* (Thunb.)DC.	Rhizomes	Hebei,china	22020905	15
Houpu	Magnoliae Officinalis Cortex	*Magnolia officinalis* Rehd.et Wils.	Root bark	Zhejiang,china	20211226	15
Chenpi	Citri Reticulatae pericarpium	*Citrus reticulata* Blanco	Cortex	Sichuan,china	20220102	15
Baizhu	Atractylodes macrocephala Koidz.	*Atractylodis Macrocephalae* Rhizoma	Root	Anhui,china	22012201	15
Fuling	Poria	*Poria cocos*（Schw.）Wolf	Dry polypite	Anhui,china	22011901	15
Zexie	Alismatis Rhizoma	*Alisma plantago-aquatica* Linn.	Tubers	Fujian,china	20220111	10
Zhuling	Polyporus	*Polyporus umbellatus* (Pers.) Fries	Dry polypite	Shanxi,china	21062401	10
Gancao	Glycyrrhizae Radix Et Rhizoma	*Glycyrrhiza uralensis* Fisch.	Root and rhizome	Inner Mongolia,china	22030701	6
Rougui	Cinnamomi Cortex	*Cinnamomum cassia* Presl	Bark	Guangxi,china	21031402	5
Shengjiang	Zingiberis Rhizoma Recens	*Zingiber officinale* Rosc.	Rhizome	Sichuan,china		10
Dazao	Jujube Fructus	*Ziziphus jujuba* Mill.	Fruit	Xinjiang,china	20220612	10
Wumei	Mume Fructus	*Prunus Mume(Sieb.)Sieb.et* Zuce.	Fruit	Sichuan,china	21091806	10
Ganjiang	Zingiberis Rhizoma	*Zingiber officinale* Rosc.	Rhizome	Sichuan,china	20220328	10

Note: the batch number is issued by BeijingTong-ren-tang Chinese medicine Co., LTD after the herbs have been tested. The plant name has been checked with http://www.worldfloraonline.org/.

Table S5 The main symptoms of CDD model

**Principal symptoms**	1. Tired and lazy, curled up in a pile<br>2. The spirit is weak, the hair color is dark, erect, easy to fall off<br>3. Loose stools and perianal soiling<br>4. Move slowly, even diagonally
**Accompanied symptoms**	1. No weight gain or slow growth<br>2. Dietary reduction
